# Phylogeography of the sand dune ant *Mycetophylax simplex* along the Brazilian Atlantic Forest coast: remarkably low mtDNA diversity and shallow population structure

**DOI:** 10.1186/s12862-015-0383-4

**Published:** 2015-06-10

**Authors:** Danon Clemes Cardoso, Maykon Passos Cristiano, Mara Garcia Tavares, Christoph D. Schubart, Jürgen Heinze

**Affiliations:** Present address: Departamento de Genética, Universidade Federal do Paraná, Setor de Ciências Biológicas, Rua Francisco H. dos Santos, s/n°, Jardim das Américas, Curitiba, Paraná 81530-000 Brazil; Departamento de Biologia Geral, Universidade Federal de Viçosa, Av. Peter Henry Rolfs, sn, Viçosa, Minas Gerais 36570-000 Brazil; Zoology/Evolutionary Biology, Universität Regensburg, Universitätstrasse 31, D-93040 Regensburg, Germany; Departamento de Biodiversidade, Evolução e Meio Ambiente, Universidade Federal de Ouro Preto, Ouro Preto, Minas Gerais 35400-000 Brazil

**Keywords:** Brazilian Atlantic Forest, *Mycetophylax simplex*, Formicidae, Neotropical region, Gene flow, Genetic structure, Sand dunes, Restinga, Sea-level changes

## Abstract

**Background:**

During past glacial periods, many species of forest-dwelling animals experienced range contractions. In contrast, species living outside such moist habitats appear to have reacted to Quaternary changes in different ways. The Atlantic Forest represents an excellent opportunity to test phylogeographic hypotheses, because it has a wide range of vegetation types, including unforested habitats covered predominantly by herbaceous and shrubby plants, which are strongly influenced by the harsh environment with strong wind and high insolation. Here, we investigated the distribution of genetic diversity in the endemic sand dune ant *Mycetophylax simplex* across its known range along the Brazilian coast, with the aim of contributing to the understanding of alternative phylogeographic patterns. We used partial sequences of the mitochondrial gene cytochrome oxidase I and nuclear gene wingless from 108 specimens and 51 specimens, respectively, to assess the phylogeography and demographic history of this species. To achieve this we performed different methods of phylogenetic and standard population genetic analyses.

**Results:**

The observed genetic diversity distribution and historical demographic profile suggests that the history of *M. simplex* does not match the scenario suggested for other Atlantic Forest species. Instead, it underwent demographic changes and range expansions during glacial periods. Our results show that *M. simplex* presents a shallow phylogeographic structure with isolation by distance among the studied populations, living in an almost panmictic population. Our coalescence approach indicates that the species maintained a stable population size until roughly 75,000 years ago, when it underwent a gradual demographic expansion that were coincident with the low sea-level during the Quaternary. Such demographic events were likely triggered by the expansion of the shorelines during the lowering of the sea level.

**Conclusions:**

Our data suggest that over evolutionary time *M. simplex* did not undergo dramatic range fragmentation, but rather it likely persisted in largely interconnected populations. Furthermore, we add an important framework about how both glacial and interglacial events could positively affect the distribution and diversification of species. The growing number of contrasting phylogeographic patterns within and among species and regions have shown that Quaternary events influenced the distribution of species in more ways than first supposed.

**Electronic supplementary material:**

The online version of this article (doi:10.1186/s12862-015-0383-4) contains supplementary material, which is available to authorized users.

## Background

Climatic fluctuations during the late Quaternary associated with the Last Glacial Maximum have had a strong impact on the current distribution of many animal and plant species worldwide. Climate change not only affected the landscape of continental areas but also the sea level, which in turn shaped coastal landscapes by forming land bridges, islands, sand coastal plains, as well as connecting and separating areas [1, 2]. All these climate-linked processes may have influenced the evolutionary history of the species, especially those inhabiting coastal areas [3, 4].

Pollen data, fossil records, and paleoclimatic data indicate that numerous taxa in the Northern hemisphere were restricted during the period of glaciation to southern or eastern refugia [5], and phylogeographic studies show how expansion from these sites has molded their distribution today [6–9]. In contrast, less is known about these phenomena in the Southern hemisphere, where the glacial refugia hypothesis only recently has been formally evaluated [10, 11]. It is presently the most frequently suggested mechanism for the current distribution of species diversity in the Brazilian Atlantic Forest (AF), the second largest Neotropical forest after the Amazon rainforest.

The AF extends more than 3300 km along the eastern coast of Brazil. It has received worldwide attention because of its high biodiversity and high level of endemism and was determined as one of the 25 world biodiversity hotspots for conservation priorities [10, 12]. The AF presents a wide range of vegetation types with conspicuous changes across landscapes, which include open habitats covered predominantly by herbaceous and shrubby plants, which develop on marine deposits. Despite an increasing number of phylogeographic studies, the knowledge about the evolutionary history of the AF is still limited and controversial [10, 13–19]. The refugia hypothesis has gained support by a growing number of phylogeographic studies that attempt to explain the high diversity in the AF [13, 15, 16]. Basically, it states that during glaciation, when the climate was drier in the Southern hemisphere, forests contracted and persisted only in moister areas, which became refugia for humidity-dependent species. By vicariant processes, these refugia promoted speciation and therefore today harbor a higher genetic diversity and endemism than areas that did not serve as refugia [1].

The retreat of forests and the changed global climate conditions may have allowed the expansion of drought-tolerant biomes [20, 21]. Species adapted to dry and open habitats in southern and eastern South America, including the Atlantic coast, may have expanded during the drier periods throughout climatic oscillations. Compared to species restricted to refugia, such species may have experienced recurrent shifts in their distribution, with populations mixing or separating from each other with the cyclical shrinking or expanding of forests. Alternatively, their distribution may have remained largely unchanged during these historical events. While several species associated with humid forest environments have been used to infer the evolutionary processes that occurred during the Quaternary in the Atlantic Forest, only a few studies have addressed organisms – exclusively plants – associated with dry environments (see [17]).

*Mycetophylax simplex* is a small fungus-growing ant (Formicidae: Myrmicinae) endemic to sand dune environments along the Brazilian coast, occurring from the southern São Paulo State to Rio Grande do Sul [22, 23]. Although this ant exhibits a wide distribution, it is restricted to specialized habitats (sand dunes), making it a good model organism to test phylogeographic scenarios for open and dry coastal environments. Thus, the aim of this study is to evaluate the genetic relationship among populations of *M. simplex* across its whole distribution and to infer how the Quaternary oscillations affected genetic diversity and structure of its populations. Based on DNA sequences of the mitochondrial gene *cytochrome oxidase subunit I* (COI) and the nuclear gene *wingless*, we aimed to test if (i) *M. simplex* underwent a persistent range and population size during the climatic oscillations of the Quaternary and if and how (ii) its demographic history was affected by the cyclic changes in the sea level.

## Methods

### Sampling and laboratory procedures

**Fig. 1:**
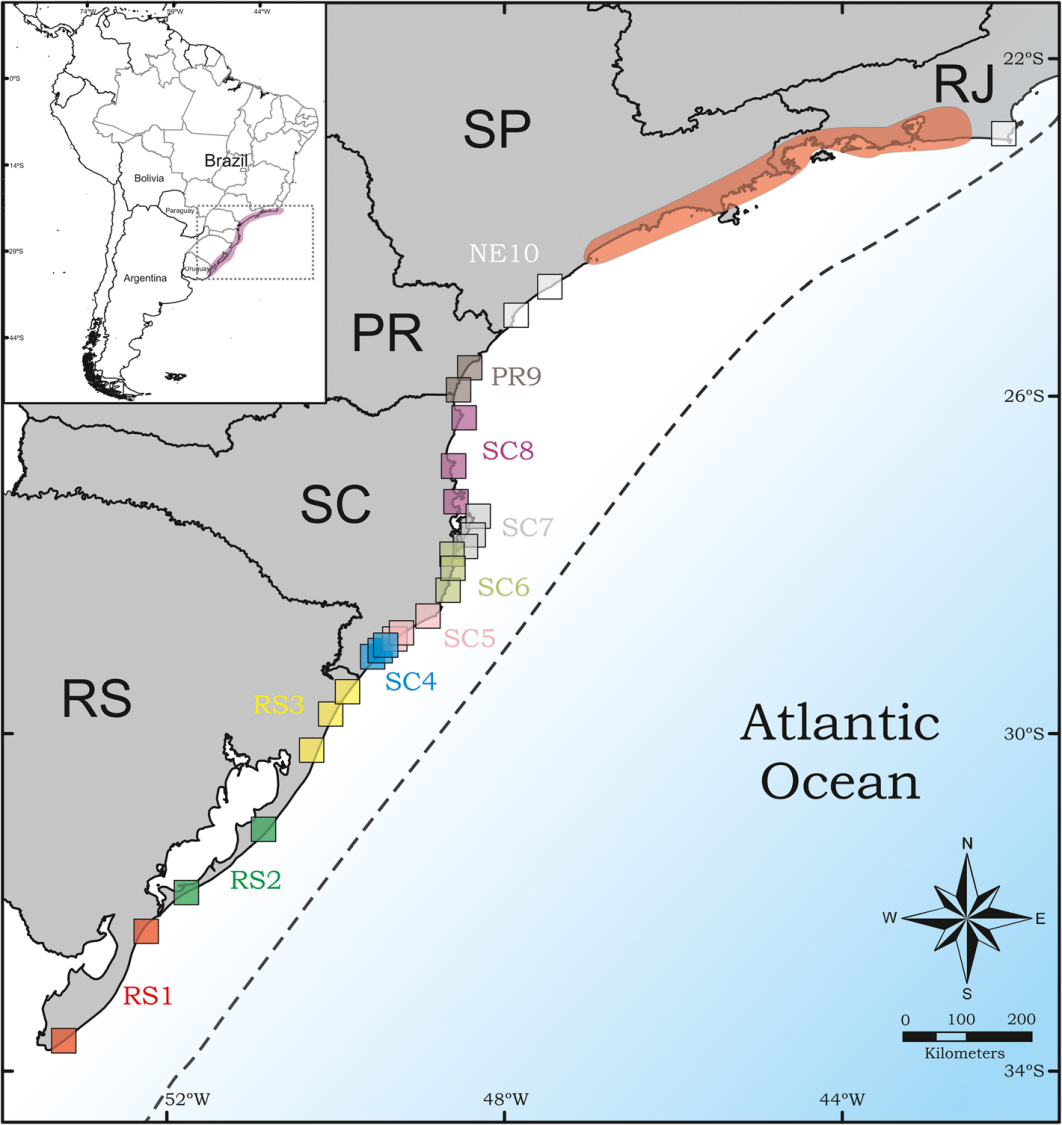
Map showing the 27 sampled localities throughout the distribution of *M simplex* on the southern and southeast Atlantic coast of Brazil. Each color square represents one population (for details see table 1). The red highlighted area on the southeast coast is the gap in the distribution of *M. simplex* where we did not find the species besides our sampling effort, and the dashed line represents the limits of the sea–level during the last glacial maximum (approximately 21 Mya). The map was produced using a GIS program with free layers available at IBGE website (www.mapas.ibge.gov.br)

A total of 108 colonies of *M. simplex,* spanning its whole distribution area, were collected from January to September 2011 (Fig. 1). The geographical references and sample size of all samples are given in Table 1. The specimens were preserved in 100 % ethanol until DNA extraction. Whole genomic DNA was extracted from one individual per colony. Genomic DNA was isolated using proteinase K digestion followed by a standard CTAB protocol [24]. Fragments of the mitochondrial gene *cytochrome oxidase subunit I* (COI) and the nuclear gene *wingless*, both known to be useful in intra-specific studies in ants (e.g. [25]), were amplified by PCR using the previously published primers LCO1490 [26] and Ben [27] for COI and Wg578F [28] and Wg1032R [29] for *wingless*. DNA-amplification was conducted in reactions with 25 μL final volume containing: MgCl_2_ (2.5 mM), buffer (10x; Promega), dNTPs (1 mM each), two primers (0.48 μM each), *Taq* polymerase (2 U of GoTaq® Flexi DNA Polymerase) and 1 μL of template DNA. PCR cycling conditions were as follows: initial denaturation for 2 min at 94 °C, then 35 cycles with 94 °C for 1 min denaturation, 50 °C (COI) or 55 °C (*wingless*) for 1 min annealing, 72 °C for 2 min extension, and a final extension at 72 °C for 7 min. PCR products were purified and sequenced by Macrogen Inc. (www.macrogen.com) in both directions using the same primers as for amplification in an ABI PRISM 3700 sequencing system. Singletons were confirmed by sequencing a second individual from the same colony when available.

**Table 1:** Population details, geographical location of the population encompassing 27 sampled localities (S = latitude, W = longitude) throughout the range distribution of *M. simplex* and its haplotype distribution

Population	Locality	Coordinate		Haplotype (number)
		S	W	
RS1	Chuí	33° 43’	53° 21’	H5(3), H22(1), H26(1)
	Cassino	32° 13’	52° 11’	H5(3), H14(1), H27(1)
RS2	São José do Norte	32° 03’	51° 59’	H5(3), H29(1)
	Mostardas	31° 07’	50° 50’	H22(2), H25(1), H28(1), H30(1)
RS3	Cidreira	30° 07’	50° 11’	H2(2), H21(1), H22(1), H23(1)
	Curumim	29° 37’	49° 56’	H2(1), H5(1), H19(1), H20(1)
	Torres	29° 21’	49° 44’	H24(1)
SC4	Bal. Arroio do Silva	29° 00’	49° 26’	H1(1), H4(1)
	Bal. Gaivota	29° 11’	49° 35’	H1(1), H4(1), H3(1), H7(1)
	Araranguá	28° 57’	49° 22’	H1(2), H7(2), H10(1)
SC5	Ilhas	28° 54’	49° 19’	H1(1), H2(1), H30(1), H31(1)
	Bal. Rincão	28° 48’	49° 12’	H1(1), H3(2), H4(1), H5(1)
	Laguna	28° 36’	48° 50’	H3(1), H29(4)
SC6	Itapirubá	28° 19’	48° 42’	H3(1), H8(1)
	Garopaba	27° 59’	48° 37’	H3(2), H9(1), H11(2)
	Pinheira	27° 50’	48° 35’	H3(1), H11(3)
SC7	Florianópolis – Moçambique	27° 29’	48° 23’	H11(2)
	Florianópolis – Joaquina	27° 37’	48° 27’	H1(1), H3(1), H11(2)
	Florianópolis – Pântano do Sul	27° 46’	48° 31’	H3(1), H11(2), H13(1), H14(1)
SC8	Gov. Celso Ramos	27° 19’	48° 32’	H27(1), H28(1)
	Navegantes	26° 51’	48° 38’	H11(4)
	São Francisco do Sul	26° 15’	48° 31’	H1(1), H3(2), H12(1), H15(1)
PR9	Guaratuba	25° 56’	48° 34’	H1(1), H3(2), H11(2)
	Pontal do Paraná	25° 40’	48° 27’	H3(1), H9(1), H11(1), H32(1)
NE10	Ilha Comprida - Cananéia	25° 02’	47° 53’	H3(2), H9(1), H11(2)
	Ilha Comprida - Iguape	24° 42’	47° 28’	H3(2), H11(3)
	Cabo Frio	22° 54’	42° 02’	H3(1), H6(1)

### Analysis

The chromatograms of each gene were evaluated and edited separately using the program Consed [30]. Afterwards, they were analyzed by translation into amino acids using the program MEGA 5.0 [31] in order to check for indels and premature stop codons. Since *wingless* did not show intraspecific variation throughout the sampled populations (51 specimens from same populations were analyzed (GenBank accession numbers: KP939178-KP939228), see Additional file 1: Appendix S1), subsequent analysis was conducted only with the mitochondrial gene COI. The examination of additional nuclear genes (*long-wave rhodopsin* (GenBank accession numbers: KC964627-KC964632) and *abdominal A* (data not show)) also failed to reveal intraspecific variability (for primers see [28]). Sequence variation was analyzed with the software MEGA 5.0, and diversity parameters, including nucleotide diversity (π) and haplotype diversity (*h*), were computed with DNASP 5 [32] for each population and for the complete dataset. To ensure reliable estimates of regional differentiation and diversity and to allow appropriate statistical analysis, geographically and ecologically close locations (neighboring beaches and/or beaches inside large rivers basins) were pooled in order to obtain ten populations with sample sizes with of at least eight colonies (in Table 1 sampling localities belonging to the same population are shown in the same colors in Fig. 1).

We used Bayesian Inference with MrBayes 3.2 [33] to infer phylogenetic relationships among the haplotypes of *M. simplex* and to assess the monophyletic status of this species. We selected the model of sequence evolution that best fit our dataset using the Akaike’s information criterion (AIC) implemented in MrModeltest 2.3 [34] and used *Trachymyrmex jamaicensis* (GenBank accession number: DQ353390) and *Cyphomyrmex costatus* (JQ617535 and JQ617502) as outgroups. The Bayesian analyses consisted of two independent runs of ten million generations each, starting with a random tree and four Markov chains, sampled every 1000 generations. The convergence among runs was verified by the average standard deviation of split frequencies that had to reach < 0.01. An appropriated burn-in was determined using Tracer v1.4 [35] and a total of 25 % of the trees were excluded as burn-out prior to producing a consensus topology. Finally, this topology was presented using the Figtree v. 1.3.1. software [36].

In order to assess the correlation between log-transformed genetic diversity and geographical distances among the sampled populations we carried out a Mantel test using the program AIS (alleles in space) [37]. The genetic differentiation among *M. simplex* populations was measured by means of F-statistics [38]. Pairwise comparisons of Φ_*ST*_ between populations were calculated using the program ARLEQUIN 3.5 [39], which allows estimating the degree of gene flow among populations of *M. simplex*.

We also carried out a spatial analysis of molecular variance using the program SAMOVA 1.0 [40] to search for partitions of sampling sites that were genetically homogenous, but maximally differentiated from each other. Based on value *K* that needs to be optimized, this method uses simulated annealing procedures to seek the best clustering option that can be defined between groups of populations among group genetic variation coefficients (F_*CT*_). Analyses were conducted five times to check consistency for different *K* values and based on 1000 simulated annealing steps with *K* increasing from 2 to 9 as the number of considered populations in each analysis. We also carried out a second SAMOVA analysis for different *K* values increasing from 2 to 20 considering each sampling location separately (despite low within location sample size) in order to obtain the results without any a priori population pooling. Thus, we could identify the clustering of samples that yielded the largest, and most significant, F_*CT*_ for a given *K*.

The genetic distances among *M. simplex* haplotypes were reconstructed using two methods: the Median Joining network algorithm implemented in NETWORK 4.6 (http://www.fluxus-engineering.com) and the statistical parsimony procedure for phylogenetic network estimation, with 95 % criterion for a parsimonious connection applied in TCS 1.21 [41].

Trends in the demographic history of populations of *M. simplex* were investigated using three different approaches. First, under the assumption of neutrality deviations in Tajima’s and Fu’s *F*_*S*_ statistics, we tested for past population expansions. A negative value in both statistic tests would reflect either purifying selection in a population at mutation-drift equilibrium, or deviations from mutation-drift equilibrium, resulting from population expansion events. Second, we observed the distribution of pairwise nucleotide differences among haplotypes and tested the deviation from the expected mismatch distribution under sudden and spatial models by means of a generalized least-squares method and Harpending’s *h* statistics. Both analyses were carried out in Arlequin 3.5. Third, we used a Bayesian skyline plot (BSP) approach [42], implemented in BEAST 1.6.1 [43], with the aim of recovering changes in the effective population size (Ne) over time. For this, we first estimated a mutation rate for the genus *Mycetophylax* from the COI sequences and fossil calibration of the molecular phylogeny of the ant tribe Attini (see [25]). Mutation rate estimates were performed under an uncorrelated lognormal-relaxed clock model, using the model of sequence evolution GTR + I + Γ with three partitions (codons 1, 2, and 3) with a random starting tree following a Yule speciation process as a prior tree. Based on fossils corresponding to the *Cyphomyrmex rimosus* group, the root node was given a lognormal distribution with a mean of 1.6, standard deviation of 1.0 and offset of 15, as described in Mehdiabadi et al. [25]. The results from three independent runs of 50 million generations sampled every 5,000 with a burn-in of 15 million were combined in TRACER 1.4.1 and checked for adequate mixing of the MCMC chains by values of effective sample size (ESS). Finally, we used the mutation rate calculated above with our intraspecific COI dataset in order to construct the timing and magnitude of changes in the effective population size using the Bayesian skyline method [42]. This model was used to estimate the effective population size through time, as the most recent common ancestor of *M. simplex.* Therefore, the BEAST software molecular clock was set to Uncorrelated Log-normal under a Relaxed Clock and analyses were performed using the SRD06 with distinct rates of sequence evolution for each codon partition and a tree prior to coalescence (constant size) was employed. Results from three independent runs with 50 million generations each (with the initial 10 % excluded as burn-out) were combined and analyzed with Tracer 1.4.1.

## Results

### Sequence variation, phylogenetic relationships and structure

We aligned a total of 108 sequences of the gene COI. After exclusion of sections at the beginning and end of the sequences, which in several samples were not complete, the analyzed fragment had a total length of 845 nucleotides. The 32 unique haplotypes were widely distributed across the range of *Mycetophylax simplex* (GenBank accession numbers: KJ842219-KJ8423260). Of the 32 polymorphic sites found, 19 were singletons and 13 were parsimony-informative. The COI sequences had an A-T bias as in most other arthropod mitochondrial genes (see [44]) and a bias against guanidine (T: 36.2 %. C: 20.3 %, A: 31.9 % G: 11.6 %), and all nucleotide substitutions were located at the third codon position.

The Bayesian phylogenetic analyses indicated that *M. simplex* forms a well-supported monophyletic cluster relative to the outgroups (Additional file 2: Appendix S2), and that all specimens studied throughout the distribution can be considered the same species. Overall, haplotype and nucleotide diversity was 0.865 ± 0.022 and 0.00346 ± 0.00019 (mean ± SD), respectively (Table 2).

**Table 2:** Genetic diversity and neutrality tests for each population and with all populations of *M. simplex* together

Populations	Nucleotide diversity (π) (± S.D.)	Haplotype diversity (*h*) (± S.D.)	Tajima's *D*	Fu's FS
RS1	0.00189 (0.00090)	0.667 (0.163)	−1.87333 (*P =* 0.0083)	−1.11562 (*P =* 0.1609)
RS2	0.00224 (0.00054)	0.889 (0.091)	−0.6299 (*P =* 0.2859)	−2.32907 (*P =* 0.0261)
RS3	0.00276 (0.00055)	0.933 (0.077)	−1.50661 (*P =* 0.0632)	−4.46904 (*P =* 0.0025)
SC4	0.00181 (0.00024)	0.818 (0.083)	0.43329 (*P =* 0.6969)	−1.02733 (*P =* 0.1714)
SC5	0.00268 (0.00036)	0.890 (0.060)	−1.09063 (*P =* 0.1463)	−2.8844 (*P =* 0.0302)
SC6	0.00336 (0.00039)	0.709 (0.099)	1.52257 (*P =* 0.9504)	1.62676 (*P =* 0.8143)
SC7	0.00387 (0.00064)	0.709 (0.137)	1.49895 (*P =* 0.9408)	0.7727 (*P =* 0.6626)
SC8	0.00346 (0.00042)	0.873 (0.089)	1.00501 (*P =* 0.8566)	−1.68615 (*P =* 0.1229)
SC9	0.00368 (0.00059)	0.833 (0.098)	0.92757 (*P =* 0.8263)	0.12678 (*P =* 0.4978)
NE10	0.00339 (0.00038)	0.697 (0.090)	1.68302 (*P =* 0.9613)	1.85074 (*P =* 0.8387)
All populations	0.00346 (0.00019)	0.865 (0.022)	−1.47062 (*P =* 0.0422)	−21.59803 (*P =* 0.0001)

**Fig. 2:**
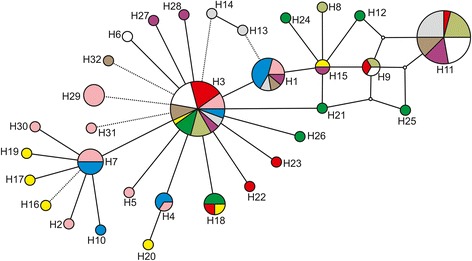
Statistical parsimony haplotype network showing the phylogenetic relationship among 32 unique haplotypes observed among ten populations of *M. simplex*. The circles are the haplotypes and their size represents their frequencies in the total sample, small and white circles are missing estimated haplotypes. Each color corresponds to the populations given in Table 1 and Fig. 1

The Median Joining haplotype network was in agreement with the statistical parsimony network. In the gene genealogy among the haplotypes (considered a 95 % threshold for the probability of a parsimonious connection being achieved) all haplotypes were joined in a single network (Fig. 2). The network did not indicate divergent clades within the genealogy of *M. simplex*. Two major haplotypes comprised 50 % of the individuals: 27.8 % of the individuals had haplotype H3, which appeared to be widespread across the species range, whereas haplotype H11, present in 22.2 % of the individuals, was almost completely restricted to the north of the species range. H11 is separated from other haplotypes by two missing intermediate haplotypes (Fig. 2). Large portions of the recovered haplotypes were singletons (Table 1), but no more than two nucleotide substitutions (mutation steps) separate neighboring haplotypes (Fig. 2).

**Fig. 3:**
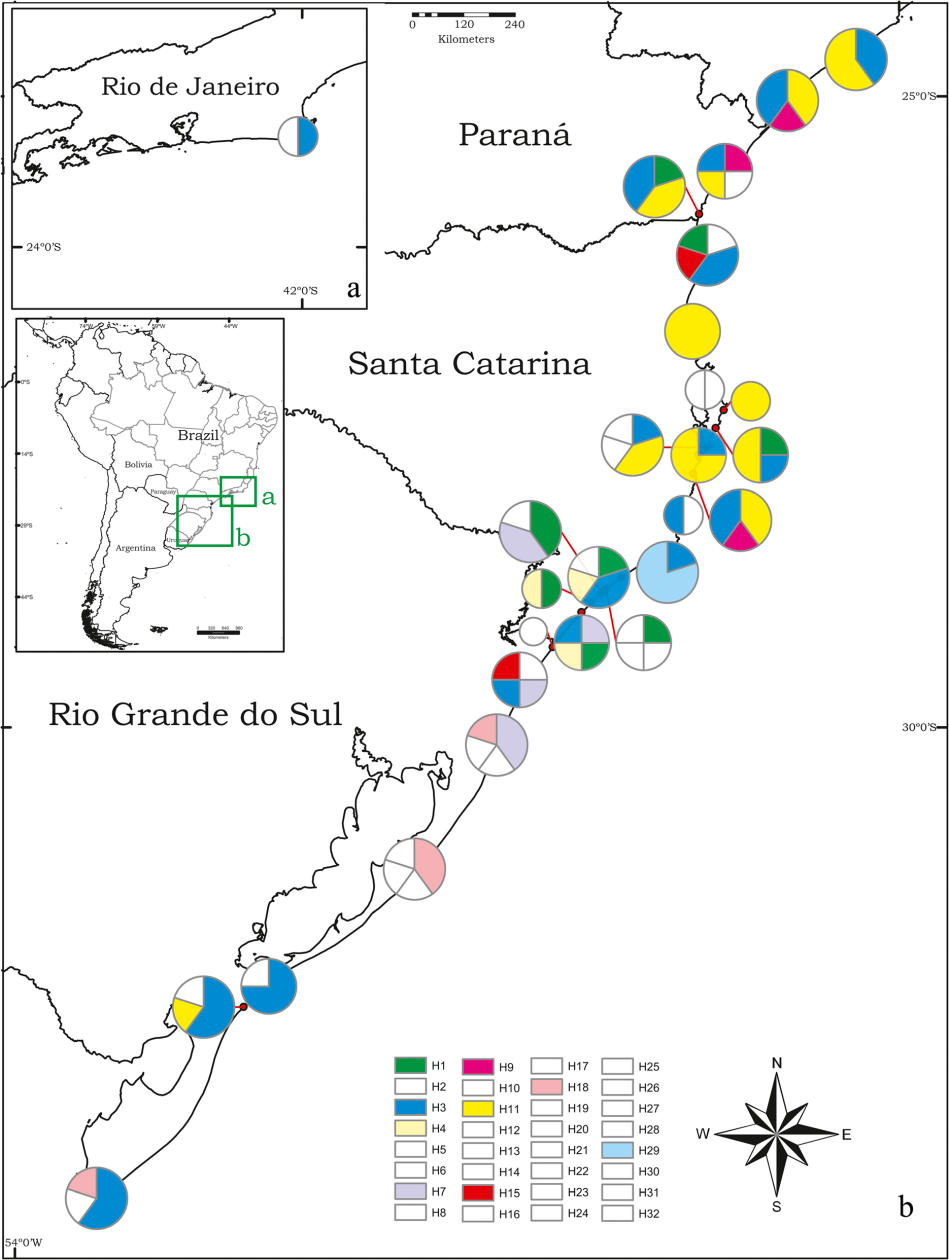
Geographical distribution of all 32 cytochrome oxidase I (COI) unique haplotypes observed across the distribution of *M. simplex* along Atlantic Brazilian coast. The circles are the haplotypes and their size represents their frequencies in the total sample, singletons were suppressed and are shown in white. Colors display frequent haplotypes distributed throughout *M. simplex* distribution along Atlantic coast. The map was produced using a GIS program with free layers available at IBGE website (www.mapas.ibge.gov.br). The colors of haplotypes do not refer to the previous figures

There was no clear association between genetic structure and geography concerning haplotypes, since several haplotypes were widespread within the range of *M. simplex* (Figs. 2 and 3). However, pairwise Φ_*ST*_ comparisons between populations indicated a certain geographic structure among the haplotypes of *M. simplex*. Populations from the southern edge of the distribution (RS1, RS2) did not show significant genetic differentiation and Φ_*ST*_ values were low (Table 3), suggesting the absence of a considerable barrier to gene flow. The same homogeneity was observed in populations from the north (SC6 – NE10). However, gene flow between these northern and southern populations appears to be restricted, which leads to the slight geographic structure observed in the distribution of *M. simplex* haplotypes likely due to isolation-by-distance in a one-dimensional distribution on the coast. Considering the non-significant Φ_*ST*_ values, the haplotypes of *M. simplex* cluster in three major population groups: southern populations (RS1 and RS2), central-eastern populations (RS3, SC4, and SC5), and northern populations (SC6 – NE10) (Table 3).

**Table 3:** - Φ_*ST*_ values for pairwise comparisons between population of *M. simplex* (lower left) and *p* values (upper right)

	**RS1**	**RS2**	**RS3**	**SC4**	**SC5**	**SC6**	**SC7**	**SC8**	**PR9**	**NE10**
**RS1**	-	0.54618	0.01228	0.04633	0.18008	0.01792	0.01594	0.04841	0.08653	0.0492
**RS2**	0.01856*	-	0.01683	0.00515	0.0302	0.02624	0.01465	0.04742	0.09554	0.0592
**RS3**	**0.11411**	**0.13078**	-	0.27888	0.02287	0.00386	0.00337	0.00485	0.01297	0.00941
**SC4**	**0.09502**	**0.1619**	0.01451**	-	0.08455	0.00356	0.00297	0.00752	0.01733	0.01129
**SC5**	0.02839	**0.093**	0.10675**	0.07119**	-	0.00069	0.0004	0.00168	0.00713	0.00614
**SC6**	**0.27381**	**0.24143**	**0.34481**	**0.34975**	**0.30795**	-	0.78299	0.92516	0.6338	0.83912
**SC7**	**0.29673**	**0.27747**	**0.36158**	**0.35894**	**0.32158**	0.05903***	-	0.59489	0.43421	0.47352
**SC8**	**0.19818**	**0.17864**	**0.28682**	**0.26863**	**0.24282**	0.06991***	0.03956***	-	0.93931	0.77794
**PR9**	0.13621	0.12338	**0.23448**	**0.21937**	**0.1883**	0.06591***	0.0298***	0.08068***	-	0.94852
**NE10**	**0.18098**	**0.16623**	**0.27856**	**0.27675**	**0.23074**	0.07182***	0.03535***	0.06859***	0.0855***	-

Population names are given in the Table 1. The colors show the shallow phylogeographic structure found: southern populations (*), central-eastern population (**) and northern populations (***)

Bold values are significant at *P* < 0.05

**Fig. 4:**
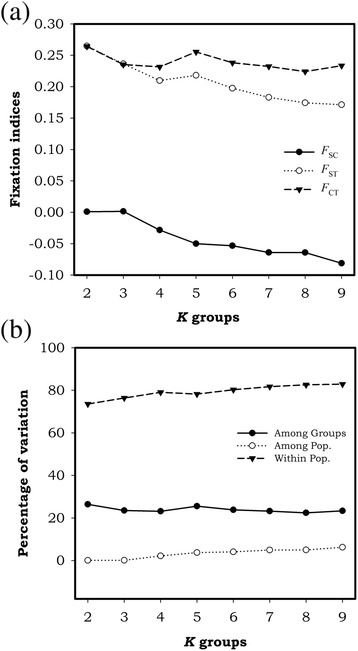
Spatial analysis of molecular variance - SAMOVA of populations of *M. simplex*. **a** Fixation indices calculated and **b** percentage of genetic variation explained by each hierarchical level for the best grouping option for each pre-specified *K* groups

In line with these results, the Mantel test showed that genetic and geographic distance are slightly correlated (r = 0.075, *p* = 0.0007), suggesting a weak degree of isolation by distance. The spatial analysis of molecular variance implemented in SAMOVA was not able to identify possible breaks among populations. All parameters varied only little (Fig. 4) and none of them suggested a better explanation for the genetic structure of *M. simplex*. F_CT_ values differed slightly from each other (0.22 - 0.26) and did not show any trend of increasing or decreasing with *K* (potential subdivisions across populations). Besides, the major proportion of variance was found within populations, ranging from 76 % to 83 % (Fig. 4b). Likewise, the second SAMOVA analysis with individual sampling sites without any a priori pooling of populations was not able to detect a best grouping scenario or possible breaks among sites (data not shown).

### Demographic history

**Fig. 5:**
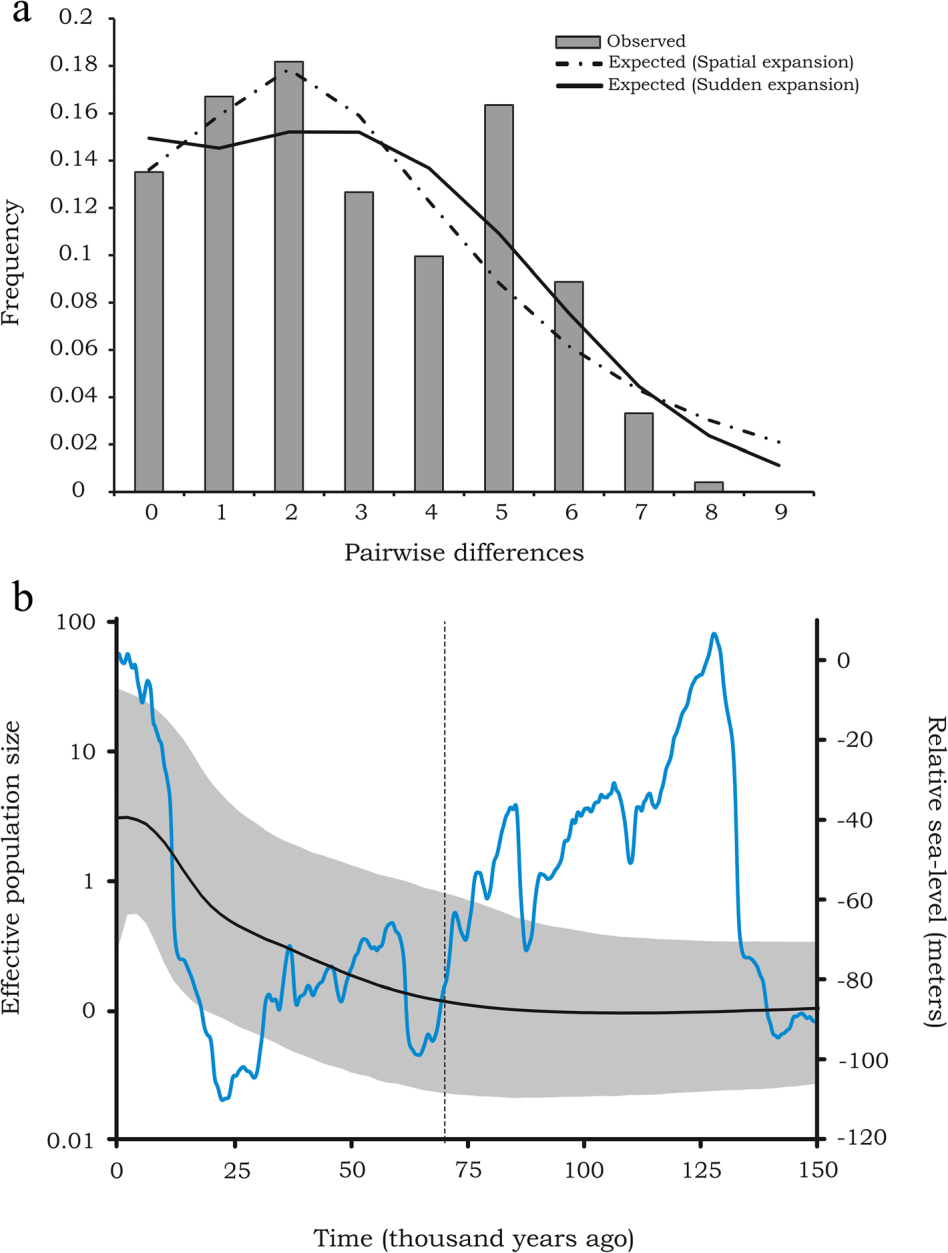
Demographic history of *M. simplex* and relative sea level. **a** Pairwise mismatch distribution of the mtDNA sequences for total dataset. *M. simplex* presented a bimodal distribution, but did not reject the spatial expansion model (SDD = 0.0226 *p =* 0.3803). **b** Bayesian skyline plot showing the historical demography and complete reconstruction of female effective population size fluctuations throughout the time of *M. simplex*. Black line represents median estimation and the grey area the upper and lower 95 % confident intervals. Dashed line indicates the beginning of demographic expansion. Blue line shows the sea-level during the last 150,000 years during the Quaternary (from [66])

As a shallow phylogeographic structure was observed in *M. simplex* populations, we conducted a historical demography analysis for the complete set of haplotypes. The neutrality tests allowed identifying some statistical significant signatures for expanding population for both tests. According to the extremely negative and significant values of Tajima’s *D* (−1.47062, *p* = 0.0422) and Fu’s *F*_*S*_ (−21.59803 *p* = 0.0001), haplotype frequencies differed from those expected for a neutral population. Yet, the mismatch distribution of pairwise nucleotide differences between haplotypes was bimodal (Fig. 5a) as expected for populations at demographic equilibrium. However, none of the models of population expansion could be rejected. Harpending’s raggedness *h* statistic (*R*_2_) and the sum of square differences (SSD) supported a close fit to the observed distribution under a pure demographic expansion model (SDD = 0.00754, *p* = 0.62760; *R*_2_ = 0.01864, *p* = 0.86400) and under a sudden spatial expansion model (SSD = 0.00905, *p* = 0.40100; *R*_2_ = 0.01864, *p* = 0.84750).

The time to the most recent common ancestor (tMRCA) for all *M. simplex* haplotypes was estimated at 0.197 Mya (with 95 % highest posterior density of 0.07-0.3559). The analysis of the Bayesian Skyline Plot provided an additional strong support for the evidence of past population expansion (Fig. 5b). The results suggested that *M. simplex* has undergone a long-term period of demographic population stability since the tMRCA lasting from ~ 70,000 years ago (Fig. 5b, dashed line). We could observe that the expansion had a second increasing cline around 25,000 years ago, corroborating the observed mismatch distribution (Fig. 5a).

## Discussion

The impact of Quaternary climatic fluctuations and geological events on the biodiversity of the Brazilian Atlantic Forest has been extensively discussed based on studies on the genetic structure of forest dweller species. Phylogeographic studies of AF species often show a clear division between northern and southern populations (e.g. [13, 14]), indicating that rivers may act as physical barriers to gene flow [13] and that species were restricted to fragmented forest refugia during glacial periods [10, 14, 15].

Here, we contribute to this discussion by exploring the phylogeography of the ant *M. simplex*, which occurs in drier, sandy coastal plain habitats along the Atlantic coast in Southern Brazil. In contrast to the above-mentioned studies on forest species (e.g. [14, 15]), our data did not reveal a strong phylogeographic pattern throughout the whole distribution of *M. simplex*. The spatial analysis of population structure implemented by SAMOVA did not suggest a congruent grouping, and we could not identify geographical barriers to gene flow. In contrast to other organisms, large bodies of water, such as rivers or the estuarine complex of the Paranaguá Bay, apparently do not impair the gene flow among the northern populations, and the populations between Florianópolis (SC) and “Ilha Comprida” (SP) were not genetically different. In sum, our results suggest that populations of *M. simplex* are not strongly isolated from one another and that genetic bottlenecks have been rare in the past. The phylogeographic pattern of *M. simplex* is probably explained best by an enduring persistence of its populations along the seashore of the Atlantic coast and unrestricted gene flow along the coast.

However, our results should be viewed with some caution. As the examined nuclear markers were invariable and not informative our conclusions are based solely on mitochondrial sequence data. Thus, such conclusions might in general be biased to some extent due the mode of inheritance, mutation rate and effective population size of mitochondrial markers rather than accurately reflecting history. Besides, results can be affected by natural selection [45].

Tropical forests (including the humid Atlantic Forest) were reduced during the Quaternary ice ages, imposing the contraction of species distribution and vicariant process [1, 6]. Yet, the drier and cooler environment during the ice ages promoted the expansion of open scrub environments in southern Brazil, including the sand dune areas along the Atlantic coast [20, 46]. Such dunes are the current habitat of *M. simplex* and the absence of a strong phylogeographic structure is therefore in agreement with the geomorphologic history of this area, suggesting that *M. simplex* may not have experienced shifts in its distribution. This result is consistent with phylogeographic patterns reported for a coastal orchid species with a similar distribution range [17]. Together, these results suggest that coastal lowland species may have had rather stable distributions during the past climatic oscillations. Similar patterns have been reported for other sand dune or coastal species worldwide [47–50].

Pairwise Φ_*ST*_ analysis allowed us to identify some restrictions to gene flow among populations from the three major geographical regions (Table 3). This might be a result of some degree of isolation by distance among the populations of *M. simplex*. Indeed, genetic and geographic distances correlated weakly (see Results). Besides, more frequent haplotypes show a wide geographic distribution (e.g., H3 and H11). This may reflect an ongoing dispersal along the coasts after LGM that continues until today. Alternatively, recurrent gene flow among nearby populations may have deleted a possible signal of the early history of this ant species.

Pleistocene sea levels fluctuated considerably through the glacial and interglacial periods during the Quaternary. During the Pleistocene, the coastal plain of the southern region of South America was larger than it is now [51, 52]. Palaeo-geomorphological studies showed that the sea-level was about 120 m lower than present (Figs. 1 and 5b), leading to an elongation of the coast-line limits to ~100 km [20, 53]. The ocean regression resulted in the enlargement of the coastal lowland. The availability of new areas for *M. simplex* may have led to the expansion of its populations. We found genetic signatures that supported this hypothesis. The negative and highly significant Fu’s *F*_*S*_ and Tajimas’s *D* showed departures from neutrality. Hence, the mismatch distribution detected imprints of both sudden and spatial demographic expansions in the past population of *M. simplex*. The historical demographic reconstruction based on a Bayesian Skyline Plot showed that *M. simplex* underwent a stable demographic period shifting to a persistent growth of effective population size in recent periods. Population growth started around 70,000 years ago and displays a slight increase around 25,000 years ago. These results indicate that the processes underlying the current diversity of *M. simplex* occurred during the middle-Pleistocene and intensified under different climatic and geological events imposed by the Last Glacial Maximum (LGM), at the Late-Pleistocene. We cannot exclude, however, other potential scenarios due the limitations of analyses based on a single locus. The historical scenario of population growth of *M. simplex* starting during LGM could be a result of over-interpretation of BSP [54], and signals about other population cycles could have been erased by population declines during the demographic history of this ant [54]. It is possible that *M. simplex* populations underwent a demographic contraction during LGM and then expanded thereafter, with the contraction not being clearly evident in the BSP.

Nevertheless, historical expansions in the demography of *M. simplex* populations would be in agreement with two events of lower historic sea-levels, i.e., the historic demographic expansion of *M. simplex* might have started and persisted during the major period of sea-level fall (see Fig. 5b). Later, when the sea level rose to its actual status, *M. simplex* populations could have persisted in areas related to Pleistocene barriers. These barriers might have been sand deposits that were initiated to be accumulated since the Last Interglacial around 120 Mya [2].

The cooler climatic conditions during the Pleistocene (lower around 3–7 °C less than today) may have not been a constraint for the distribution of *M. simplex,* since this species seems to be intolerant to warm temperatures (Cardoso, pers. obs.). Today, *M. simplex* has an almost exclusively subtropical distribution, under the Tropic of Capricorn (23° S), except one residual population at Cabo Frio beach in Rio de Janeiro state. The present distribution again suggests the important role of ocean transgression. Its northernmost limit coincides with the major enlargement of the coastal lowland in Southern Brazil, from southern Chuí in Rio Grande do Sul State to Cabo Frio in Rio de Janeiro State (Fig. 1) [53] (see also [1]). The resulting new land bridges may have enabled migration of many organisms across the latitudinal gradient by forming sand corridors or sand inlands due to coastal deposits.

Bodies of water resulting from the regression and transgression of the sea apparently did not present an obstacle for flying insects and did not impair the dispersion of ants [55]. Sandy beach corridors and islands were formed during at least three phases, with sea levels similar to the current level until 6,500 years ago [53]. Thus, *M. simplex* may have had enough time to expand its range in an almost panmictic population. Sexuals of fungus-growing ants typically mate during nuptial flights. Winged male and female sexuals leave their natal colonies. After mating, young queens then disperse downwind on the ground to establish a new colony [56]. The dynamics and timing of nuptial flights and subsequent dispersal differ among species, but in general, the mating biology of ants remains remarkably unstudied [57, 58]. Leaf-cutter ants with large queens, such as *Atta* and *Acromyrmex*, appear to have low dispersal distances [59, 60]. In contrast, queens with small body size, such as those of *M. simplex*, can stay in the air for considerable time and might fly for kilometers [61]. In this way, *M. simplex* might be able to maintain gene flow and dispersal along the coast.

The absence of *M. simplex* between southern São Paulo and Cabo Frio could be explained by three non-exclusive hypotheses: (i) Holocene marine transgression drowned the suitable habits in that *M. simplex* occurred; (ii) such open habitats were removed by the expansion of the forests towards the seashore, which is currently covered by the tropical Atlantic rain Forest; (iii) competition with congeneric species.

Overall, Rio de Janeiro is characterized by a rocky coastline with little development of transitional sedimentary coastal plains, due to the proximity of the mountainous relief of Serra do Mar [62]. As an effect of Post Glacial Marine Transgression, the sea submerged the majority of sandy beach ridges; removing *M. simplex* from this portion of Brazilian coastline. They might have survived in the region of Cabo Frio because of the presence of Pleistocene sand deposits that might have acted as refugia during the marine transgressive events [17]. Cabo Frio and surrounding regions are the only area in the south-eastern Brazilian coast with such aeolian dunes, a typical habitat of *M. simplex* [62].

Furthermore, post-glacial AF expansion together with the rise of the sea level may have led to the shrinkage of sand dune habitats between 23° and 24° S [63]. Finally, *M. simplex* co-occurs with *M. conformis* north of São Paulo State. Both species nest near the seashore and use debris from the sparse vegetation to grow their fungus gardens (see [64] for nesting details). *M. conformis* seems to tolerate a broader range of ecological conditions nesting in sunny and shaded environments of sand dunes and the workers are active during the day and night (Cardoso, pers. obs.). Yet, *M. simplex* exclusively nests in sunny sand dune places and its workers are active only during dusk and overnight [65]. All three factors may have reduced the local density of *M. simplex* and finally led to its extinction in the area between southern São Paulo and Cabo Frio. Indeed, *M. conformis* was found living in the northern range where *M. simplex* was not found, beside our sampling effort.

## Conclusions

Phylogeographic studies regarding species associated with open and dry environments have attracted much less attention compared to the number of studies using organisms associated with forests as a study model. Identifying explicit phylogeographical patterns and the factors underpinning genetic structure are reasonably difficult, particularly for those species inhabiting historic and dynamic regions, such as coastal sand dunes. Our findings indicate that *M. simplex* presents an evolutionary history consistent with shifts in the sea-level and changes in the distribution of dry vegetation in southeastern Brazil. The diversification and expansion started in the mid-Pleistocene, during which major climatic changes occurred worldwide. Our results are in agreement with other studies with sand dune species that indicate expansion during glacial periods, but is contrasting with others, suggesting that a single and wide model of Quaternary effects on the diversification and distribution of species is unrealistic.

## Availability of supporting data

All datasets supporting of this article are provided with Additional files. All gene sequences obtained in this study were deposited in the GenBank database under the following accession numbers: *wingless* (KP939178-KP939228) and *cytochrome oxidase subunit* I (KJ842219-KJ8423260).

## Additional files

Additional file 1: Appendix S1. Sequence alignments for 372 base pairs of *Mycetophylax simplex* wingless gene from 51 specimens (GenBank accession numbers: KP939178-KP939228). The 51 individuals sequenced here are the same individuals sequenced for COI gene. The alignment included none variable sites and none parsimony informative sites. When phased in the DnaSP program the data show only eight segregating sites and none number of polymorphic segregating sites with more than two variants. Additional file 2: Appendix S2. Bayesian phylogenetic consensus tree of COI gene sequences of *M. simplex* haplotypes and outgroups. The numbers at branches are Bayesian posterior probabilities (P.P.). Tree branches are proportional to mutational events sampled in sequences alignment (scale bar).

## Abbreviations

AF: Brazilian Atlantic Forest
AIC: Akaike’s Information Criterion
BSP: Bayesian skyline plot
COI: Cytochrome oxidase subunit I
CTAB: Cetyltrimethyl ammonium bromide
DNA: Deoxyribonucleic acid
dNTPs: Deoxynucleotides
ESS: Effective sample sizes
Γ: Gamma distribution
GIS: Geographic information system
GTR: General time reversible model
I: Proportion of invariable sites
IBGE: Brazilian Institute of Geography and Statistics
LGM: Last Glacial Maximum
MCMC: Markov Chain Monte Carlo
mtDNA: Mitochondrial DNA
Mya: Million years ago
PCR: Polymerase Chain Reaction
PP: Posterior probability
SAMOVA: Spatial analysis of molecular variance
SD: Standard deviation
SSD: Sum of square differences
tMRCA: The most recent common ancestor
