# Erratum to: Phylogeography of the sand dune ant *Mycetophylax simplex* along the Brazilian Atlantic Forest coast: remarkably low mtDNA diversity and shallow population structure

**DOI:** 10.1186/s12862-015-0444-8

**Published:** 2015-09-15

**Authors:** Danon Clemes Cardoso, Maykon Passos Cristiano, Mara Garcia Tavares, Christoph D. Schubart, Jürgen Heinze

**Affiliations:** Present address: Departamento de Genética, Universidade Federal do Paraná, Setor de Ciências Biológicas, Rua Francisco H. dos Santos, s/n°, Jardim das Américas, Curitiba, 81530-000 Paraná Brazil; Departamento de Biologia Geral, Universidade Federal de Viçosa, Av. Peter Henry Rolfs, sn, Viçosa, 36570-000 Minas Gerais Brazil; Zoology/Evolutionary Biology, Universität Regensburg, Universitätstrasse 31, Regensburg, D-93040 Germany; Departamento de Biodiversidade, Evolução e Meio Ambiente, Universidade Federal de Ouro Preto, Ouro Preto, 35400-000 Minas Gerais Brazil

## Erratum

The original version of this article [1] unfortunately contained a mistake. The presentation of Tables two and three (Tables 1 and 2 here) were incorrect in the PDF and HTML versions of this article. The corrected Tables two and three (Tables 1 and 2 here) are given below.

**Table 1:** Genetic diversity and neutrality tests for each population and with all populations of M. simplex together

Populations	Nucleotide diversity (π) (± S.D.)	Haplotype diversity (*h*) (± S.D.)	Tajima's *D*	Fu's FS
RS1	0.00189 (0.00090)	0.667 (0.163)	−1.87333 (*P =* 0.0083)	−1.11562 (*P =* 0.1609)
RS2	0.00224 (0.00054)	0.889 (0.091)	−0.6299 (*P =* 0.2859)	−2.32907 (*P =* 0.0261)
RS3	0.00276 (0.00055)	0.933 (0.077)	−1.50661 (*P =* 0.0632)	−4.46904 (*P =* 0.0025)
SC4	0.00181 (0.00024)	0.818 (0.083)	0.43329 (*P =* 0.6969)	−1.02733 (*P =* 0.1714)
SC5	0.00268 (0.00036)	0.890 (0.060)	−1.09063 (*P =* 0.1463)	−2.8844 (*P =* 0.0302)
SC6	0.00336 (0.00039)	0.709 (0.099)	1.52257 (*P =* 0.9504)	1.62676 (*P =* 0.8143)
SC7	0.00387 (0.00064)	0.709 (0.137)	1.49895 (*P =* 0.9408)	0.7727 (*P =* 0.6626)
SC8	0.00346 (0.00042)	0.873 (0.089)	1.00501 (*P =* 0.8566)	−1.68615 (*P =* 0.1229)
SC9	0.00368 (0.00059)	0.833 (0.098)	0.92757 (*P =* 0.8263)	0.12678 (*P =* 0.4978)
NE10	0.00339 (0.00038)	0.697 (0.090)	1.68302 (*P =* 0.9613)	1.85074 (*P =* 0.8387)
All populations	0.00346 (0.00019)	0.865 (0.022)	−1.47062 (*P =* 0.0422)	−21.59803 (*P =* 0.0001)

Cardoso *et al.*

Cardoso *et al. BMC Evolutionary Biology* 2015 **15**:106 doi:10.1186/s12862-015-0383-4

**Table 2:** **-** Φ_***ST***_ values for pairwise comparisons between population of *M. simplex* (lower left) and *p* values (upper right)

	**RS1**	**RS2**	**RS3**	**SC4**	**SC5**	**SC6**	**SC7**	**SC8**	**PR9**	**NE10**
**RS1**	-	0.54618	0.01228	0.04633	0.18008	0.01792	0.01594	0.04841	0.08653	0.0492
**RS2**	0.01856^a^	-	0.01683	0.00515	0.0302	0.02624	0.01465	0.04742	0.09554	0.0592
**RS3**	**0.11411**	**0.13078**	-	0.27888	0.02287	0.00386	0.00337	0.00485	0.01297	0.00941
**SC4**	**0.09502**	**0.1619**	0.01451^b^	-	0.08455	0.00356	0.00297	0.00752	0.01733	0.01129
**SC5**	0.02839	**0.093**	**0.10675** ^**b**^	0.07119^b^	-	0.00069	0.0004	0.00168	0.00713	0.00614
**SC6**	**0.27381**	**0.24143**	**0.34481**	**0.34975**	**0.30795**	-	0.78299	0.92516	0.6338	0.83912
**SC7**	**0.29673**	**0.27747**	**0.36158**	**0.35894**	**0.32158**	0.05903^c^	-	0.59489	0.43421	0.47352
**SC8**	**0.19818**	**0.17864**	**0.28682**	**0.26863**	**0.24282**	0.06991^c^	0.03956^c^	-	0.93931	0.77794
**PR9**	0.13621	0.12338	**0.23448**	**0.21937**	**0.1883**	0.06591^c^	0.0298^c^	0.08068^c^	-	0.94852
**NE10**	**0.18098**	**0.16623**	**0.27856**	**0.27675**	**0.23074**	0.07182^c^	0.03535^c^	0.06859^c^	0.0855^c^	-

Population names are given in the Table 1. The colors show the shallow phylogeographic structure found: southern populations (^a^), central-eastern population (^b^) and northern populations (^c^)

Bold values are significant at *P* < 0.05

Cardoso *et al.*

Cardoso *et al. BMC Evolutionary Biology* 2015 **15**:106 doi:10.1186/s12862-015-0383-4

The original article has been updated to reflect this change.
