# Supplementary material for: Phylogeography of the sand dune ant Mycetophylax simplex along the Brazilian Atlantic Forest coast: remarkably low mtDNA diversity and shallow population structure
Source: BMC Evol Biol. 2015 Jun 10;15:106. doi: 10.1186/s12862-015-0383-4 (PMC4460702; doi:10.1186/s12862-015-0383-4)
Supplement: Additional file 1: Appendix S1. — Sequence alignments for 372 base pairs of Mycetophylax simplex wingless gene from 51 specimens (GenBank accession numbers: KP939178-KP939228). The 51 individuals sequenced here are the same individuals sequenced for COI gene. The alignment included none variable sites and none parsimony informative sites. When phased in the DnaSP program the data show only eight segregating sites and none number of polymorphic segregating sites with more than two variants. [file 12862_2015_383_MOESM1_ESM.pdf]

Appendix S1: Sequence alignments for 372 base pairs of Mycetophylax simplex wingless gene from 51 specimens (GenBank accession numbers: KP939178-KP939228). The 51 individuals sequenced here are the same individuals sequenced for COI gene. The alignment included none variable sites and none parsimony informative sites. When phased in the DnaSP program the data show only eight segregating sites and none number of polymorphic segregating sites with more than two variants.

	1									10										20										30										40										50										60
mx001	G	T	T	T	C	G	A	C	G	G	G	G	C	T	T	C	T	C	G	G	G	T	A	A	T	G	G	T	C	A	G	T	A	A	C	T	C	G	G	A	A	C	G	A	G	C	G	C	G	C	G	T	T	A	C	C	A	A	C	A
mx087	G	T	T	T	C	G	A	C	G	G	G	G	C	T	T	C	T	C	G	G	G	T	A	A	T	G	G	T	C	A	G	T	A	A	C	T	C	G	G	A	A	C	G	A	G	C	G	C	G	C	G	T	T	A	C	C	A	A	C	A
mx035	G	T	T	T	C	G	A	C	G	G	G	G	C	T	T	C	T	C	G	G	G	T	A	A	T	G	G	T	C	A	G	T	A	A	C	T	C	G	G	W	R	C	G	W	G	C	G	C	G	C	G	T	T	A	C	C	A	A	C	A
mx004	G	T	T	T	C	G	A	C	G	G	G	G	C	T	T	C	T	C	G	G	G	T	A	A	T	G	G	T	C	A	G	T	A	A	C	T	C	G	G	W	R	C	G	W	G	C	G	C	G	C	G	T	T	A	C	C	A	A	C	A
mx008	G	T	T	T	C	G	A	C	G	G	G	G	C	T	T	C	T	C	G	G	G	T	A	A	T	G	G	T	C	A	G	T	A	A	C	T	C	G	G	A	A	C	G	A	G	C	G	C	G	C	G	T	T	A	C	C	A	A	C	A
mx042	G	T	T	T	C	G	A	C	G	G	G	G	C	T	T	C	T	C	G	G	G	T	A	A	T	G	G	T	C	A	G	T	A	A	C	T	C	G	G	A	A	C	G	A	G	C	G	C	G	C	G	T	T	A	C	C	A	A	C	A
mx002	G	T	T	T	C	G	A	C	G	G	G	G	C	T	T	C	T	C	G	G	G	T	A	A	T	G	G	T	C	A	G	T	A	A	C	T	C	G	G	A	A	C	G	A	G	C	G	C	G	C	G	T	T	A	C	C	A	A	C	A
mx053	G	T	T	T	C	G	A	C	G	G	G	G	C	T	T	C	T	C	G	G	G	T	A	A	T	G	G	T	C	A	G	T	A	A	C	T	C	G	G	W	R	C	G	W	G	C	G	C	G	C	G	T	T	A	C	C	A	A	C	A
mx109	G	T	T	T	C	G	A	C	G	G	G	G	C	T	T	C	T	C	G	G	G	T	A	A	T	G	G	T	C	A	G	T	A	A	C	T	C	G	G	A	A	C	G	A	G	C	G	C	G	C	G	T	T	A	C	C	A	A	C	A
mx110	G	T	T	T	C	G	A	C	G	G	G	G	C	T	T	C	T	C	G	G	G	T	A	A	T	G	G	T	C	A	G	T	A	A	C	T	C	G	G	A	A	C	G	A	G	C	G	C	G	C	G	T	T	A	C	C	A	A	C	A
mx013	G	T	T	T	C	G	A	C	G	G	G	G	C	T	T	C	T	C	G	G	G	T	A	A	T	G	G	T	C	A	G	T	A	A	C	T	C	G	G	A	A	C	G	A	G	C	G	C	G	C	G	T	T	A	C	C	A	A	C	A
mx034	G	T	T	T	C	G	A	C	G	G	G	G	C	T	T	C	T	C	G	G	G	T	A	A	T	G	G	T	C	A	G	T	A	A	C	T	C	G	G	A	A	C	G	A	G	C	G	C	G	C	G	T	T	A	C	C	A	A	C	A
mx020	G	T	T	T	C	G	A	C	G	G	G	G	C	T	T	C	T	C	G	G	G	T	A	A	T	G	G	T	C	A	G	T	A	A	C	T	C	G	G	A	A	C	G	A	G	C	G	C	G	C	G	T	T	A	C	C	A	A	C	A
mx048	G	T	T	T	C	G	A	C	G	G	G	G	C	T	T	C	T	C	G	G	G	T	A	A	T	G	G	T	C	A	G	T	A	A	C	T	C	G	G	A	A	C	G	A	G	C	G	C	G	C	G	T	T	A	C	C	A	A	C	A
mx045	G	T	T	T	C	G	A	C	G	G	G	G	C	T	T	C	T	C	G	G	G	T	A	A	T	G	G	T	C	A	G	T	A	A	C	T	C	G	G	A	A	C	G	A	G	C	G	C	G	C	G	T	T	A	C	C	A	A	C	A
mx025	G	T	T	T	C	G	A	C	G	G	G	G	C	T	T	C	T	C	G	G	G	T	A	A	T	G	G	T	C	A	G	T	A	A	C	T	C	G	G	A	A	C	G	A	G	C	G	C	G	C	G	T	T	A	C	C	A	A	C	A
mx024	G	T	T	T	C	G	A	C	G	G	G	G	C	T	T	C	T	C	G	G	G	T	A	A	T	G	G	T	C	A	G	T	A	A	C	T	C	G	G	A	A	C	G	A	G	C	G	C	G	C	G	T	T	A	C	C	A	A	C	A
mx044	G	T	T	T	C	G	A	C	G	G	G	G	C	T	T	C	T	C	G	G	G	T	A	A	T	G	G	T	C	A	G	T	A	A	C	T	C	G	G	A	A	C	G	A	G	C	G	C	G	C	G	T	T	A	C	C	A	A	C	A
mx049	G	T	T	T	C	G	A	C	G	G	G	G	C	T	T	C	T	C	G	G	G	T	A	A	T	G	G	T	C	A	G	T	A	A	C	T	C	G	G	A	A	C	G	A	G	C	G	C	G	C	G	T	T	A	C	C	A	A	C	A
mx050	G	T	T	T	C	G	A	C	G	G	G	G	C	T	T	C	T	C	G	G	G	T	A	A	T	G	G	T	C	A	G	T	A	A	C	T	C	G	G	A	A	C	G	A	G	C	G	C	G	C	G	T	T	A	C	C	A	A	C	A
mx051	G	T	T	T	C	G	A	C	G	G	G	G	C	T	T	C	T	C	G	G	G	T	A	A	T	G	G	T	C	A	G	T	A	A	C	T	C	G	G	A	A	C	G	A	G	C	G	C	G	C	G	T	T	A	C	C	A	A	C	A
mx055	G	T	T	T	C	G	A	C	G	G	G	G	C	T	T	C	T	C	G	G	G	T	A	A	T	G	G	T	C	A	G	T	A	A	C	T	C	G	G	A	A	C	G	A	G	C	G	C	G	C	G	T	T	A	C	C	A	A	C	A
mx111	G	T	T	T	C	G	A	C	G	G	G	G	C	T	T	C	T	C	G	G	G	T	A	A	T	G	G	T	C	A	G	T	A	A	C	T	C	G	G	A	A	C	G	A	G	C	G	C	G	C	G	T	T	A	C	C	A	A	C	A
mx069	G	T	T	T	C	G	A	C	G	G	G	G	C	T	T	C	T	C	G	G	G	T	A	A	T	G	G	T	C	A	G	T	A	A	C	T	C	G	G	W	R	C	G	W	G	C	G	C	G	C	G	T	T	A	C	C	A	A	C	A
mx062	G	T	T	T	C	G	A	C	G	G	G	G	C	T	T	C	T	C	G	G	G	T	A	A	T	G	G	T	C	A	G	T	A	A	C	T	C	G	G	W	R	C	G	W	G	C	G	C	G	C	G	T	T	A	C	C	A	A	C	A
mx112	G	T	T	T	C	G	A	C	G	G	G	G	C	T	T	C	T	C	G	G	G	T	A	A	T	G	G	T	C	A	G	T	A	A	C	T	C	G	G	A	A	C	G	A	G	C	G	C	G	C	G	T	T	A	C	C	A	A	C	A
mx065	G	T	T	T	C	G	A	C	G	G	G	G	C	T	T	C	T	C	G	G	G	T	A	A	T	G	G	T	C	A	G	T	A	A	C	T	C	G	G	W	R	C	G	W	G	C	G	C	G	C	G	T	T	A	C	C	A	A	C	A
mx074	G	T	T	T	C	G	A	C	G	G	G	G	C	T	T	C	T	C	G	G	G	T	A	A	T	G	G	T	C	A	G	T	A	A	C	T	C	G	G	A	A	C	G	A	G	C	G	C	G	C	G	T	T	A	C	C	A	A	C	A
mx084	G	T	T	T	C	G	A	C	G	G	G	G	C	T	T	C	T	C	G	G	G	T	A	A	T	G	G	T	C	A	G	T	A	A	C	T	C	G	G	W	R	C	G	W	G	C	G	C	G	C	G	T	T	A	C	C	A	A	C	A
mx066	G	T	T	T	C	G	A	C	G	G	G	G	C	T	T	C	T	C	G	G	G	T	A	A	T	G	G	T	C	A	G	T	A	A	C	T	C	G	G	W	R	C	G	W	G	C	G	C	G	C	G	T	T	A	C	C	A	A	C	A
mx072	G	T	T	T	C	G	A	C	G	G	G	G	C	T	T	C	T	C	G	G	G	T	A	A	T	G	G	T	C	A	G	T	A	A	C	T	C	G	G	W	R	C	G	W	G	C	G	C	G	C	G	T	T	A	C	C	A	A	C	A
mx086	G	T	T	T	C	G	A	C	G	G	G	G	C	T	T	C	T	C	G	G	G	T	A	A	T	G	G	T	C	A	G	T	A	A	C	T	C	G	G	W	R	C	G	W	G	C	G	C	G	C	G	T	T	A	C	C	A	A	C	A
mx091	G	T	T	T	C	G	A	C	G	G	G	G	C	T	T	C	T	C	G	G	G	T	A	A	T	G	G	T	C	A	G	T	A	A	C	T	C	G	G	A	A	C	G	A	G	C	G	C	G	C	G	T	T	A	C	C	A	A	C	A
mx113	G	T	T	T	C	G	A	C	G	G	G	G	C	T	T	C	T	C	G	G	G	T	A	A	T	G	G	T	C	A	G	T	A	A	C	T	C	G	G	W	R	C	G	W	G	C	G	C	G	C	G	T	T	A	C	C	A	A	C	A
mx067	G	T	T	T	C	G	A	C	G	G	G	G	C	T	T	C	T	C	G	G	G	T	A	A	T	G	G	T	C	A	G	T	A	A	C	T	C	G	G	A	A	C	G	A	G	C	G	C	G	C	G	T	T	A	C	C	A	A	C	A
mx014	G	T	T	T	C	G	A	C	G	G	G	G	C	T	T	C	T	C	G	G	G	T	A	A	T	G	G	T	C	A	G	T	A	A	C	T	C	G	G	A	A	C	G	A	G	C	G	C	G	C	G	T	T	A	C	C	A	A	C	A
mx007	G	T	T	T	C	G	A	C	G	G	G	G	C	T	T	C	T	C	G	G	G	T	A	A	T	G	G	T	C	A	G	T	A	A	C	T	C	G	G	A	A	C	G	A	G	C	G	C	G	C	G	T	T	A	C	C	A	A	C	A
mx017	G	T	T	T	C	G	A	C	G	G	G	G	C	T	T	C	T	C	G	G	G	T	A	A	T	G	G	T	C	A	G	T	A	A	C	T	C	G	G	A	A	C	G	A	G	C	G	C	G	C	G	T	T	A	C	C	A	A	C	A
mx026	G	T	T	T	C	G	A	C	G	G	G	G	C	T	T	C	T	C	G	G	G	T	A	A	T	G	G	T	C	A	G	T	A	A	C	T	C	G	G	W	R	C	G	W	G	C	G	C	G	C	G	T	T	A	C	C	A	A	C	A
mx060	G	T	T	T	C	G	A	C	G	G	G	G	C	T	T	C	T	C	G	G	G	T	A	A	T	G	G	T	C	A	G	T	A	A	C	T	C	G	G	A	A	C	G	A	G	C	G	C	G	C	G	T	T	A	C	C	A	A	C	A
mx054	G	T	T	T	C	G	A	C	G	G	G	G	C	T	T	C	T	C	G	G	G	T	A	A	G	G	G	T	C	A	G	T	A	A	C	T	C	G	G	A	A	C	G	A	G	C	G	C	G	C	G	T	T	A	C	C	A	A	C	A
mx076	G	T	T	T	C	G	A	C	G	G	G	G	C	T	T	C	T	C	G	G	G	T	A	A	T	G	G	T	C	A	G	T	A	A	C	T	C	G	G	A	A	C	G	A	G	C	G	C	G	C	G	T	T	A	C	C	A	A	C	A
mx085	G	T	T	T	C	G	A	C	G	G	G	G	C	T	T	C	T	C	G	G	G	T	A	A	T	G	G	T	C	A	G	T	A	A	C	T	C	G	G	A	A	C	G	A	G	C	G	C	G	C	G	T	T	A	C	C	A	A	C	A
mx114	G	T	T	T	C	G	A	C	G	G	G	G	C	T	T	C	T	C	G	G	G	T	A	A	T	G	G	T	C	A	G	T	A	A	C	T	C	G	G	A	A	C	G	A	G	C	G	C	G	C	G	T	T	A	C	C	A	A	C	A
mx098	G	T	T	T	C	G	A	C	G	G	G	G	C	T	T	C	T	C	G	G	G	T	A	A	T	G	G	T	C	A	G	T	A	A	C	T	C	G	G	A	A	C	G	A	G	C	G	C	G	C	G	T	T	A	C	C	A	A	C	A
mx092	G	T	T	T	C	G	A	C	G	G	G	G	C	T	T	C	T	C	G	G	G	T	A	A	T	G	G	T	C	A	G	T	A	A	C	T	C	G	G	A	A	C	G	A	G	C	G	C	G	C	G	T	T	A	C	C	A	A	C	A
mx099	G	T	T	T	C	G	A	C	G	G	G	G	C	T	T	C	T	C	G	G	G	T	A	A	T	G	G	T	C	A	G	T	A	A	C	T	C	G	G	W	R	C	G	W	G	C	G	C	G	C	G	T	T	A	C	C	A	A	C	A
mx096	G	T	T	T	C	G	A	C	G	G	G	G	C	T	T	C	T	C	G	G	G	T	A	A	T	G	G	T	C	A	G	T	A	A	C	T	C	G	G	A	A	C	G	A	G	C	G	C	G	C	G	T	T	A	C	C	A	A	C	A
mx097	G	T	T	T	C	G	A	C	G	G	G	G	C	T	T	C	T	C	G	G	G	T	A	A	T	G	G	T	C	A	G	T	A	A	C	T	C	G	G	A	A	C	G	A	G	C	G	C	G	C	G	T	T	A	C	C	A	A	C	A
mx095	G	T	T	T	C	G	A	C	G	G	G	G	C	T	T	C	T	C	G	G	G	T	A	A	T	G	G	T	C	A	G	T	A	A	C	T	C	G	G	A	A	C	G	A	G	C	G	C	G	C	G	T	T	A	C	C	A	A	C	A
mx094	G	T	T	T	C	G	A	C	G	G	G	G	C	T	T	C	T	C	G	G	G	T	A	A	T	G	G	T	C	A	G	T	A	A	C	T	C	G	G	A	A	C	G	A	G	C	G	C	G	C	G	T	T	A	C	C	A	A	C	A

Continued

	61									70										80										90										100										110										120
mx001	A	T	G	C	C	A	T	C	A	C	C	A	G	C	A	A	C	T	C	G	G	C	T	A	G	C	A	A	T	T	C	C	G	T	A	C	A	T	C	A	G	C	A	T	C	G	T	G	A	T	G	G	T	C	T	C	G	G	G	C
mx087	A	T	G	C	C	A	T	C	A	C	C	A	G	C	A	A	C	T	C	G	G	C	T	A	G	C	A	A	T	T	C	C	G	T	A	C	A	T	C	A	G	C	A	T	C	G	T	G	A	T	G	G	T	C	T	C	G	G	G	C
mx035	A	T	G	C	C	A	T	C	A	C	C	A	G	C	A	A	C	T	C	G	G	C	T	A	G	C	A	A	T	T	C	C	G	T	A	C	A	T	C	A	G	C	A	T	C	G	T	G	A	T	G	G	T	C	T	C	G	G	G	C
mx004	A	T	G	C	C	A	T	C	A	C	C	A	G	C	A	A	C	T	C	R	G	C	T	A	G	C	A	A	T	T	C	C	G	T	A	C	A	T	C	A	G	C	A	T	C	G	T	G	A	T	G	G	T	C	T	C	G	G	G	C
mx008	A	T	G	C	C	A	T	C	A	C	C	A	G	C	A	A	C	T	C	G	G	C	T	A	G	C	A	A	T	T	C	C	G	T	A	C	A	T	C	A	G	C	A	T	C	G	T	G	A	T	G	G	T	C	T	C	G	G	G	C
mx042	A	T	G	C	C	A	T	C	A	C	C	A	G	C	A	A	C	T	C	G	G	C	T	A	G	C	A	A	T	T	C	C	G	T	A	C	A	T	C	A	G	C	A	T	C	G	T	G	A	T	G	G	T	C	T	C	G	G	G	C
mx002	A	T	G	C	C	A	T	C	A	C	C	A	G	C	A	A	C	T	C	G	G	C	T	A	G	C	A	A	T	T	C	C	G	T	A	C	A	T	C	A	G	C	A	T	C	G	T	G	A	T	G	G	T	C	T	C	G	G	G	C
mx053	A	T	G	C	C	A	T	C	A	C	C	A	G	C	A	A	C	T	C	G	G	C	T	A	G	C	A	A	T	T	C	C	G	T	A	C	A	T	C	A	G	C	A	T	C	G	T	G	A	T	G	G	T	C	T	C	G	G	G	C
mx109	A	T	G	C	C	A	T	C	A	C	C	A	G	C	A	A	C	T	C	G	G	C	T	A	G	C	A	A	T	T	C	C	G	T	A	C	A	T	C	A	G	C	A	T	C	G	T	G	A	T	G	G	T	C	T	C	G	G	G	C
mx110	A	T	G	C	C	A	T	C	A	C	C	A	G	C	A	A	C	T	C	G	G	C	T	A	G	C	A	A	T	T	C	C	G	T	A	C	A	T	C	A	G	C	A	T	C	G	T	G	A	T	G	G	T	C	T	C	G	G	G	C
mx013	A	T	G	C	C	A	T	C	A	C	C	A	G	C	A	A	C	T	C	G	G	C	T	A	G	C	A	A	T	T	C	C	G	T	A	C	A	T	C	A	G	C	A	T	C	G	T	G	A	T	G	G	T	C	T	C	G	G	G	C
mx034	A	T	G	C	C	A	T	C	A	C	C	A	G	C	A	A	C	T	C	G	G	C	T	A	G	C	A	A	T	T	C	C	G	T	A	C	A	T	C	A	G	C	A	T	C	G	T	G	A	T	G	G	T	C	T	C	G	G	G	C
mx020	A	T	G	C	C	A	T	C	A	C	C	A	G	C	A	A	C	T	C	G	G	C	T	A	G	C	A	A	T	T	C	C	G	T	A	C	A	T	C	A	G	C	A	T	C	G	T	G	A	T	G	G	T	C	T	C	G	G	G	C
mx048	A	T	G	C	C	A	T	C	A	C	C	A	G	C	A	A	C	T	C	G	G	C	T	A	G	C	A	A	T	T	C	C	G	T	A	C	A	T	C	A	G	C	A	T	C	G	T	G	A	T	G	G	T	C	T	C	G	G	G	C
mx045	A	T	G	C	C	A	T	C	A	C	C	A	G	C	A	A	C	T	C	G	G	C	T	A	G	C	A	A	T	T	C	C	G	T	A	C	A	T	C	A	G	C	A	T	C	G	T	G	A	T	G	G	T	C	T	C	G	G	G	C
mx025	A	T	G	C	C	A	T	C	A	C	C	A	G	C	A	A	C	T	C	G	G	C	T	A	G	C	A	A	T	T	C	C	G	T	A	C	A	T	C	A	G	C	A	T	C	G	T	G	A	T	G	G	T	C	T	C	G	G	G	C
mx024	A	T	G	C	C	A	T	C	A	C	C	A	G	C	A	A	C	T	C	G	G	C	T	A	G	C	A	A	T	T	C	C	G	T	A	C	A	T	C	A	G	C	A	T	C	G	T	G	A	T	G	G	T	C	T	C	G	G	G	C
mx044	A	T	G	C	C	A	T	C	A	C	C	A	G	C	A	A	C	T	C	G	G	C	T	A	G	C	A	A	T	T	C	C	G	T	A	C	A	T	C	A	G	C	A	T	C	G	T	G	A	T	G	G	T	C	T	C	G	G	G	C
mx049	A	T	G	C	C	A	T	C	A	C	C	A	G	C	A	A	C	T	C	G	G	C	T	A	G	C	A	A	T	T	C	C	G	T	A	C	A	T	C	A	G	C	A	T	C	G	T	G	A	T	G	G	T	C	T	C	G	G	G	C
mx050	A	T	G	C	C	A	T	C	A	C	C	A	G	C	A	A	C	T	C	G	G	C	T	A	G	C	A	A	T	T	C	C	G	T	A	C	A	T	C	A	G	C	A	T	C	G	T	G	A	T	G	G	T	C	T	C	G	G	G	C
mx051	A	T	G	C	C	A	T	C	A	C	C	A	G	C	A	A	C	T	C	G	G	C	T	A	G	C	A	A	T	T	C	C	G	T	A	C	A	T	C	A	G	C	A	T	C	G	T	G	A	T	G	G	T	C	T	C	G	G	G	C
mx055	A	T	G	C	C	A	T	C	A	C	C	A	G	C	A	A	C	T	C	G	G	C	T	A	G	C	A	A	T	T	C	C	G	T	A	C	A	T	C	A	G	C	A	T	C	G	T	G	A	T	G	G	T	C	T	C	G	G	G	C
mx111	A	T	G	C	C	A	T	C	A	C	C	A	G	C	A	A	C	T	C	G	G	C	T	A	G	C	A	A	T	T	C	C	G	T	A	C	A	T	C	A	G	C	A	T	C	G	T	G	A	T	G	G	T	C	T	C	G	G	G	C
mx069	A	T	G	C	C	A	T	C	A	C	C	A	G	C	A	A	C	T	C	G	G	C	T	A	G	C	A	A	T	T	C	C	G	T	A	C	A	T	C	A	G	C	A	T	C	G	T	G	A	T	G	G	T	C	T	C	G	G	G	C
mx062	A	T	G	C	C	A	T	C	A	C	C	A	G	C	A	A	C	T	C	R	G	C	T	A	G	C	A	A	T	T	C	C	G	T	A	C	A	T	C	A	G	C	A	T	C	G	T	G	A	T	G	G	T	C	T	C	G	G	G	C
mx112	A	T	G	C	C	A	T	C	A	C	C	A	G	C	A	A	C	T	C	R	G	C	T	A	G	C	A	A	T	T	C	C	G	T	A	C	A	T	C	A	G	C	A	T	C	G	T	G	A	T	G	G	T	C	T	C	G	G	G	C
mx065	A	T	G	C	C	A	T	C	A	C	C	A	G	C	A	A	C	T	C	G	G	C	T	A	G	C	A	A	T	T	C	C	G	T	A	C	A	T	C	A	G	C	A	T	C	G	T	G	A	T	G	G	T	C	T	C	G	G	G	C
mx074	A	T	G	C	C	A	T	C	A	C	C	A	G	C	A	A	C	T	C	G	G	C	T	A	G	C	A	A	T	T	C	C	G	T	R	C	A	T	C	A	G	C	A	T	C	G	T	G	A	T	G	G	T	C	T	C	G	G	G	C
mx084	A	T	G	C	C	A	T	C	A	C	C	A	G	C	A	A	C	T	C	G	G	C	T	A	G	C	A	A	T	T	C	C	G	T	A	C	A	T	C	A	G	C	A	T	C	G	T	G	A	T	G	G	T	C	T	C	G	G	G	C
mx066	A	T	G	C	C	A	T	C	A	C	C	A	G	C	A	A	C	T	C	G	G	C	T	A	G	C	A	A	T	T	C	C	G	T	A	C	A	T	C	A	G	C	A	T	C	G	T	G	A	T	G	G	T	C	T	C	G	G	G	C
mx072	A	T	G	C	C	A	T	C	A	C	C	A	G	C	A	A	C	T	C	G	G	C	T	A	G	C	A	A	T	T	C	C	G	T	A	C	A	T	C	A	G	C	A	T	C	G	T	G	A	T	G	G	T	C	T	C	G	G	G	C
mx086	A	T	G	C	C	A	T	C	A	C	C	A	G	C	A	A	C	T	C	R	G	C	T	A	G	C	A	A	T	T	C	C	G	T	A	C	A	T	C	A	G	C	A	T	C	G	T	G	A	T	G	G	T	C	T	C	G	G	G	C
mx091	A	T	G	C	C	A	T	C	A	C	C	A	G	C	A	A	C	T	C	G	G	C	T	A	G	C	A	A	T	T	C	C	G	T	A	C	A	T	C	A	G	C	A	T	C	G	T	G	A	T	G	G	T	C	T	C	G	G	G	C
mx113	A	T	G	C	C	A	T	C	A	C	C	A	G	C	A	A	C	T	C	G	G	C	T	A	G	C	A	A	T	T	C	C	G	T	A	C	A	T	C	A	G	C	A	T	C	G	T	G	A	T	G	G	T	C	T	C	G	G	G	C
mx067	A	T	G	C	C	A	T	C	A	C	C	A	G	C	A	A	C	T	C	G	G	C	T	A	G	C	A	A	T	T	C	C	G	T	A	C	A	T	C	A	G	C	A	T	C	G	T	G	A	T	G	G	T	C	T	C	G	G	G	C
mx014	A	T	G	C	C	A	T	C	A	C	C	A	G	C	A	A	C	T	C	G	G	C	T	A	G	C	A	A	T	T	C	C	G	T	A	C	A	T	C	A	G	C	A	T	C	G	T	G	A	T	G	G	T	C	T	C	G	G	G	C
mx007	A	T	G	C	C	A	T	C	A	C	C	A	G	C	A	A	C	T	C	G	G	C	T	A	G	C	A	A	T	T	C	C	G	T	A	C	A	T	C	A	G	C	A	T	C	G	T	G	A	T	G	G	T	C	T	C	G	G	G	C
mx017	A	T	G	C	C	A	T	C	A	C	C	A	G	C	A	A	C	T	C	G	G	C	T	A	G	C	A	A	T	T	C	C	G	T	A	C	A	T	C	A	G	C	A	T	C	G	T	G	A	T	G	G	T	C	T	C	G	G	G	C
mx026	A	T	G	C	C	A	T	C	A	C	C	A	G	C	A	A	C	T	C	G	G	C	T	A	G	C	A	A	T	T	C	C	G	T	A	C	A	T	C	A	G	C	A	T	C	G	T	G	A	T	G	G	T	C	T	C	G	G	G	C
mx060	A	T	G	C	C	A	T	C	A	C	C	A	G	C	A	A	C	T	C	G	G	C	T	A	G	C	A	A	T	T	C	C	G	T	A	C	A	T	C	A	G	C	A	T	C	G	T	G	A	T	G	G	T	C	T	C	G	G	G	C
mx054	A	T	G	C	C	A	T	C	A	C	C	A	G	C	A	A	C	T	C	G	G	C	T	A	G	C	A	A	T	T	C	C	G	T	A	C	A	T	C	A	G	C	A	T	C	G	T	G	A	T	G	G	T	C	T	C	G	G	G	C
mx076	A	T	G	C	C	A	T	C	A	C	C	A	G	C	A	A	C	T	C	G	G	C	T	A	G	C	A	A	T	T	C	C	G	T	A	C	A	T	C	A	G	C	A	T	C	G	T	G	A	T	G	G	T	C	T	C	G	G	G	C
mx085	A	T	G	C	C	A	T	C	A	C	C	A	G	C	A	A	C	T	C	G	G	C	T	A	G	C	A	A	T	T	C	C	G	T	A	C	A	T	C	A	G	C	A	T	C	G	T	G	A	T	G	G	T	C	T	C	G	G	G	C
mx114	A	T	G	C	C	A	T	C	A	C	C	A	G	C	A	A	C	T	C	G	G	C	T	A	G	C	A	A	T	T	C	C	G	T	R	C	A	T	C	A	G	C	A	T	C	G	T	G	A	T	G	G	T	C	T	C	G	G	G	C
mx098	A	T	G	C	C	A	T	C	A	C	C	A	G	C	A	A	C	T	C	G	G	C	T	A	G	C	A	A	T	T	C	C	G	T	A	C	A	T	C	A	G	C	A	T	C	G	T	G	A	T	G	G	T	C	T	C	G	G	G	C
mx092	A	T	G	C	C	A	T	C	A	C	C	A	G	C	A	A	C	T	C	G	G	C	T	A	G	C	A	A	T	T	C	C	G	T	A	C	A	T	C	A	G	C	A	T	C	G	T	G	A	T	G	G	T	C	T	C	G	G	G	C
mx099	A	T	G	C	C	A	T	C	A	C	C	A	G	C	A	A	C	T	C	G	G	C	T	A	G	C	A	A	T	T	C	C	G	T	A	C	A	T	C	A	G	C	A	T	C	G	T	G	A	T	G	G	T	C	T	C	G	G	G	C
mx096	A	T	G	C	C	A	T	C	A	C	C	A	G	C	A	A	C	T	C	G	G	C	T	A	G	C	A	A	T	T	C	C	G	T	A	C	A	T	C	A	G	C	A	T	C	G	T	G	A	T	G	G	T	C	T	C	G	G	G	C
mx097	A	T	G	C	C	A	T	C	A	C	C	A	G	C	A	A	C	T	C	G	G	C	T	A	G	C	A	A	T	T	C	C	G	T	A	C	A	T	C	A	G	C	A	T	C	G	T	G	A	T	G	G	T	C	T	C	G	G	G	C
mx095	A	T	G	C	C	A	T	C	A	C	C	A	G	C	A	A	C	T	C	G	G	C	T	A	G	C	A	A	T	T	C	C	G	T	A	C	A	T	C	A	G	C	A	T	C	G	T	G	A	T	G	G	T	C	T	C	G	G	G	C
mx094	A	T	G	C	C	A	T	C	A	C	C	A	G	C	A	A	C	T	C	G	G	C	T	A	G	C	A	A	T	T	C	C	G	T	A	C	A	T	C	A	G	C	A	T	C	G	T	G	A	T	G	G	T	C	T	C	G	G	G	C

Continued

	121									130										140										150										160										170										180
mx001	G	T	C	G	G	C	A	G	C	G	C	T	A	C	A	A	T	T	T	C	C	A	G	C	T	G	A	A	A	C	C	T	T	A	C	A	A	T	C	C	G	G	A	G	C	A	T	A	A	A	C	C	G	C	C	C	G	G	G	C
mx087	G	T	C	G	G	C	A	G	C	G	C	T	A	C	A	A	T	T	T	C	C	A	G	C	T	G	A	A	A	C	C	T	T	A	C	A	A	T	C	C	G	G	W	G	C	A	T	A	A	A	C	C	G	C	C	C	G	G	G	C
mx035	G	T	C	G	G	C	A	G	C	G	C	T	A	C	A	A	T	T	T	C	C	A	G	C	T	G	A	A	A	C	C	T	T	A	C	A	A	T	C	C	G	G	W	G	C	A	T	A	A	A	C	C	G	C	C	C	G	G	G	C
mx004	G	T	C	G	G	C	A	G	C	G	C	T	A	C	A	A	T	T	T	C	C	A	G	C	T	G	A	A	A	C	C	T	T	A	C	A	A	T	C	C	G	G	A	G	C	A	T	A	A	A	C	C	G	C	C	C	G	G	G	C
mx008	G	T	C	G	G	C	A	G	C	G	C	T	A	C	A	A	T	T	T	C	C	A	G	C	T	G	A	A	A	C	C	T	T	A	C	A	A	T	C	C	G	G	A	G	C	A	T	A	A	A	C	C	G	C	C	Y	G	G	G	C
mx042	G	T	C	G	G	C	A	G	C	G	C	T	A	C	A	A	T	T	T	C	C	A	G	C	T	G	A	A	A	C	C	T	T	A	C	A	A	T	C	C	G	G	W	G	C	A	T	A	A	A	C	C	G	C	C	C	G	G	G	C
mx002	G	T	C	G	G	C	A	G	C	G	C	T	A	C	A	A	T	T	T	C	C	A	G	C	T	G	A	A	A	C	C	T	T	A	C	A	A	T	C	C	G	G	A	G	C	A	T	A	A	A	C	C	G	C	C	C	G	G	G	C
mx053	G	T	C	G	G	C	A	G	C	G	C	T	A	C	A	A	T	T	T	C	C	A	G	C	T	G	A	A	A	C	C	T	T	A	C	A	A	T	C	C	G	G	W	G	C	A	T	A	A	A	C	C	G	C	C	C	G	G	G	C
mx109	G	T	C	G	G	C	A	G	C	G	C	T	A	C	A	A	T	T	T	C	C	A	G	C	T	G	A	A	A	C	C	T	T	A	C	A	A	T	C	C	G	G	A	G	C	A	T	A	A	A	C	C	G	C	C	C	G	G	G	C
mx110	G	T	C	G	G	C	A	G	C	G	C	T	A	C	A	A	T	T	T	C	C	A	G	C	T	G	A	A	A	C	C	T	T	A	C	A	A	T	C	C	G	G	A	G	C	A	T	A	A	A	C	C	G	C	C	C	G	G	G	C
mx013	G	T	C	G	G	C	A	G	C	G	C	T	A	C	A	A	T	T	T	C	C	A	G	C	T	G	A	A	A	C	C	T	T	A	C	A	A	T	C	C	G	G	A	G	C	A	T	A	A	A	C	C	G	C	C	C	G	G	G	C
mx034	G	T	C	G	G	C	A	G	C	G	C	T	A	C	A	A	T	T	T	C	C	A	G	C	T	G	A	A	A	C	C	T	T	A	C	A	A	T	C	C	G	G	A	G	C	A	T	A	A	A	C	C	G	C	C	C	G	G	G	C
mx020	G	T	C	G	G	C	A	G	C	G	C	T	A	C	A	A	T	T	T	C	C	A	G	C	T	G	A	A	A	C	C	T	T	A	C	A	A	T	C	C	G	G	A	G	C	A	T	A	A	A	C	C	G	C	C	C	G	G	G	C
mx048	G	T	C	G	G	C	A	G	C	G	C	T	A	C	A	A	T	T	T	C	C	A	G	C	T	G	A	A	A	C	C	T	T	A	C	A	A	T	C	C	G	G	A	G	C	A	T	A	A	A	C	C	G	C	C	C	G	G	G	C
mx045	G	T	C	G	G	C	A	G	C	G	C	T	A	C	A	A	T	T	T	C	C	A	G	C	T	G	A	A	A	C	C	T	T	A	C	A	A	T	C	C	G	G	W	G	C	A	T	A	A	A	C	C	G	C	C	C	G	G	G	C
mx025	G	T	C	G	G	C	A	G	C	G	C	T	A	C	A	A	T	T	T	C	C	A	G	C	T	G	A	A	A	C	C	T	T	A	C	A	A	T	C	C	G	G	A	G	C	A	T	A	A	A	C	C	G	C	C	C	G	G	G	C
mx024	G	T	C	G	G	C	A	G	C	G	C	T	A	C	A	A	T	T	T	C	C	A	G	C	T	G	A	A	A	C	C	T	T	A	C	A	A	T	C	C	G	G	A	G	C	A	T	A	A	A	C	C	G	C	C	C	G	G	G	C
mx044	G	T	C	G	G	C	A	G	C	G	C	T	A	C	A	A	T	T	T	C	C	A	G	C	T	G	A	A	A	C	C	T	T	A	C	A	A	T	C	C	G	G	W	G	C	A	T	A	A	A	C	C	G	C	C	C	G	G	G	C
mx049	G	T	C	G	G	C	A	G	C	G	C	T	A	C	A	A	T	T	T	C	C	A	G	C	T	G	A	A	A	C	C	T	T	A	C	A	A	T	C	C	G	G	A	G	C	A	T	A	A	A	C	C	G	C	C	C	G	G	G	C
mx050	G	T	C	G	G	C	A	G	C	G	C	T	A	C	A	A	T	T	T	C	C	A	G	C	T	G	A	A	A	C	C	T	T	A	C	A	A	T	C	C	G	G	W	G	C	A	T	A	A	A	C	C	G	C	C	C	G	G	G	C
mx051	G	T	C	G	G	C	A	G	C	G	C	T	A	C	A	A	T	T	T	C	C	A	G	C	T	G	A	A	A	C	C	T	T	A	C	A	A	T	C	C	G	G	W	G	C	A	T	A	A	A	C	C	G	C	C	C	G	G	G	C
mx055	G	T	C	G	G	C	A	G	C	G	C	T	A	C	A	A	T	T	T	C	C	A	G	C	T	G	A	A	A	C	C	T	T	A	C	A	A	T	C	C	G	G	W	G	C	A	T	A	A	A	C	C	G	C	C	C	G	G	G	C
mx111	G	T	C	G	G	C	A	G	C	G	C	T	A	C	A	A	T	T	T	C	C	A	G	C	T	G	A	A	A	C	C	T	T	A	C	A	A	T	C	C	G	G	W	G	C	A	T	A	A	A	C	C	G	C	C	C	G	G	G	C
mx069	G	T	C	G	G	C	A	G	C	G	C	T	A	C	A	A	T	T	T	C	C	A	G	C	T	G	A	A	A	C	C	T	T	A	C	A	A	T	C	C	G	G	W	G	C	A	T	A	A	A	C	C	G	C	C	C	G	G	G	C
mx062	G	T	C	G	G	C	A	G	C	G	C	T	A	C	A	A	T	T	T	C	C	A	G	C	T	G	A	A	A	C	C	T	T	A	C	A	A	T	C	C	G	G	W	G	C	A	T	A	A	A	C	C	G	C	C	C	G	G	G	C
mx112	G	T	C	G	G	C	A	G	C	G	C	T	A	C	A	A	T	T	T	C	C	A	G	C	T	G	A	A	A	C	C	T	T	A	C	A	A	T	C	C	G	G	A	G	C	A	T	A	A	A	C	C	G	C	C	C	G	G	G	C
mx065	G	T	C	G	G	C	A	G	C	G	C	T	A	C	A	A	T	T	T	C	C	A	G	C	T	G	A	A	A	C	C	T	T	A	C	A	A	T	C	C	G	G	W	G	C	A	T	A	A	A	C	C	G	C	C	C	G	G	G	C
mx074	G	T	C	G	G	C	A	G	C	G	C	T	A	C	A	A	T	T	T	C	C	A	G	C	T	G	A	A	A	C	C	T	T	A	C	A	A	T	C	C	G	G	A	G	C	A	T	A	A	A	C	C	G	C	C	C	G	G	G	C
mx084	G	T	C	G	G	C	A	G	C	G	C	T	A	C	A	A	T	T	T	C	C	A	G	C	T	G	A	A	A	C	C	T	T	A	C	A	A	T	C	C	G	G	W	G	C	A	T	A	A	A	C	C	G	C	C	Y	G	G	G	C
mx066	G	T	C	G	G	C	A	G	C	G	C	T	A	C	A	A	T	T	T	C	C	A	G	C	T	G	A	A	A	C	C	T	T	A	C	A	A	T	C	C	G	G	W	G	C	A	T	A	A	A	C	C	G	C	C	C	G	G	G	C
mx072	G	T	C	G	G	C	A	G	C	G	C	T	A	C	A	A	T	T	T	C	C	A	G	C	T	G	A	A	A	C	C	T	T	A	C	A	A	T	C	C	G	G	W	G	C	A	T	A	A	A	C	C	G	C	C	C	G	G	G	C
mx086	G	T	C	G	G	C	A	G	C	G	C	T	A	C	A	A	T	T	T	C	C	A	G	C	T	G	A	A	A	C	C	T	T	A	C	A	A	T	C	C	G	G	W	G	C	A	T	A	A	A	C	C	G	C	C	C	G	G	G	C
mx091	G	T	C	G	G	C	A	G	C	G	C	T	A	C	A	A	T	T	T	C	C	A	G	C	T	G	A	A	A	C	C	T	T	A	C	A	A	T	C	C	G	G	A	G	C	A	T	A	A	A	C	C	G	C	C	C	G	G	G	C
mx113	G	T	C	G	G	C	A	G	C	G	C	T	A	C	A	A	T	T	T	C	C	A	G	C	T	G	A	A	A	C	C	T	T	A	C	A	A	T	C	C	G	G	W	G	C	A	T	A	A	A	C	C	G	C	C	Y	G	G	G	C
mx067	G	T	C	G	G	C	A	G	C	G	C	T	A	C	A	A	T	T	T	C	C	A	G	C	T	G	A	A	A	C	C	T	T	A	C	A	A	T	C	C	G	G	A	G	C	A	T	A	A	A	C	C	G	C	C	Y	G	G	G	C
mx014	G	T	C	G	G	C	A	G	C	G	C	T	A	C	A	A	T	T	T	C	C	A	G	C	T	G	A	A	A	C	C	T	T	A	C	A	A	T	C	C	G	G	A	G	C	A	T	A	A	A	C	C	G	C	C	C	G	G	G	C
mx007	G	T	C	G	G	C	A	G	C	G	C	T	A	C	A	A	T	T	T	C	C	A	G	C	T	G	A	A	A	C	C	T	T	A	C	A	A	T	C	C	G	G	A	G	C	A	T	A	A	A	C	C	G	C	C	C	G	G	G	C
mx017	G	T	C	G	G	C	A	G	C	G	C	T	A	C	A	A	T	T	T	C	C	A	G	C	T	G	A	A	A	C	C	T	T	A	C	A	A	T	C	C	G	G	A	G	C	A	T	A	A	A	C	C	G	C	C	C	G	G	G	C
mx026	G	T	C	G	G	C	A	G	C	G	C	T	A	C	A	A	T	T	T	C	C	A	G	C	T	G	A	A	A	C	C	T	T	A	C	A	A	T	C	C	G	G	W	G	C	A	T	A	A	A	C	C	G	C	C	C	G	G	G	C
mx060	G	T	C	G	G	C	A	G	C	G	C	T	A	C	A	A	T	T	T	C	C	A	G	C	T	G	A	A	A	C	C	T	T	A	C	A	A	T	C	C	G	G	A	G	C	A	T	A	A	A	C	C	G	C	C	C	G	G	G	C
mx054	G	T	C	G	G	C	A	G	C	G	C	T	A	C	A	A	T	T	T	C	C	A	G	C	T	G	A	A	A	C	C	T	T	A	C	A	A	T	C	C	G	G	W	G	C	A	T	A	A	A	C	C	G	C	C	C	G	G	G	C
mx076	G	T	C	G	G	C	A	G	C	G	C	T	A	C	A	A	T	T	T	C	C	A	G	C	T	G	A	A	A	C	C	T	T	A	C	A	A	T	C	C	G	G	W	G	C	A	T	A	A	A	C	C	G	C	C	Y	G	G	G	C
mx085	G	T	C	G	G	C	A	G	C	G	C	T	A	C	A	A	T	T	T	C	C	A	G	C	T	G	A	A	A	C	C	T	T	A	C	A	A	T	C	C	G	G	W	G	C	A	T	A	A	A	C	C	G	C	C	Y	G	G	G	C
mx114	G	T	C	G	G	C	A	G	C	G	C	T	A	C	A	A	T	T	T	C	C	A	G	C	T	G	A	A	A	C	C	T	T	A	C	A	A	T	C	C	G	G	A	G	C	A	T	A	A	A	C	C	G	C	C	C	G	G	G	C
mx098	G	T	C	G	G	C	A	G	C	G	C	T	A	C	A	A	T	T	T	C	C	A	G	C	T	G	A	A	A	C	C	T	T	A	C	A	A	T	C	C	G	G	A	G	C	A	T	A	A	A	C	C	G	C	C	C	G	G	G	C
mx092	G	T	C	G	G	C	A	G	C	G	C	T	A	C	A	A	T	T	T	C	C	A	G	C	T	G	A	A	A	C	C	T	T	A	C	A	A	T	C	C	G	G	A	G	C	A	T	A	A	A	C	C	G	C	C	C	G	G	G	C
mx099	G	T	C	G	G	C	A	G	C	G	C	T	A	C	A	A	T	T	T	C	C	A	G	C	T	G	A	A	A	C	C	T	T	A	C	A	A	T	C	C	G	G	A	G	C	A	T	A	A	A	C	C	G	C	C	C	G	G	G	C
mx096	G	T	C	G	G	C	A	G	C	G	C	T	A	C	A	A	T	T	T	C	C	A	G	C	T	G	A	A	A	C	C	T	T	A	C	A	A	T	C	C	G	G	A	G	C	A	T	A	A	A	C	C	G	C	C	C	G	G	G	C
mx097	G	T	C	G	G	C	A	G	C	G	C	T	A	C	A	A	T	T	T	C	C	A	G	C	T	G	A	A	A	C	C	T	T	A	C	A	A	T	C	C	G	G	A	G	C	A	T	A	A	A	C	C	G	C	C	C	G	G	G	C
mx095	G	T	C	G	G	C	A	G	C	G	C	T	A	C	A	A	T	T	T	C	C	A	G	C	T	G	A	A	A	C	C	T	T	A	C	A	A	T	C	C	G	G	A	G	C	A	T	A	A	A	C	C	G	C	C	C	G	G	G	C
mx094	G	T	C	G	G	C	A	G	C	G	C	T	A	C	A	A	T	T	T	C	C	A	G	C	T	G	A	A	A	C	C	T	T	A	C	A	A	T	C	C	G	G	A	G	C	A	T	A	A	A	C	C	G	C	C	C	G	G	G	C

Continued

	181								190										200										210										220										230										240
mx001	A	A	A	A	G	G	A	T	C	T	G	G	T	C	T	A	T	G	T	G	G	A	G	C	C	G	T	C	G	C	C	G	C	C	A	T	T	C	T	G	C	G	A	G	A	A	G	A	A	T	C	C	C	A	A	A	C	T	C	G
mx087	A	A	A	A	G	G	A	T	C	T	G	G	T	C	T	A	T	G	T	G	G	A	G	C	C	G	T	C	G	C	C	G	C	C	A	T	T	C	T	G	C	G	A	G	A	A	G	A	A	T	C	C	C	A	A	A	C	T	C	G
mx035	A	A	A	A	G	G	A	T	C	T	G	G	T	C	T	A	T	G	T	G	G	A	G	C	C	G	T	C	G	C	C	G	C	C	A	T	T	C	T	G	C	G	A	G	A	A	G	A	A	T	C	C	C	A	A	A	C	T	C	G
mx004	A	A	A	A	G	G	A	T	C	T	G	G	T	C	T	A	T	G	T	G	G	A	G	C	C	G	T	C	G	C	C	G	C	C	A	T	T	C	T	G	C	G	A	G	A	A	G	A	A	T	C	C	C	A	A	A	C	T	C	G
mx008	A	A	A	A	G	G	A	T	C	T	G	G	T	C	T	A	T	G	T	G	G	A	G	C	C	G	T	C	G	C	C	G	C	C	A	T	T	C	T	G	C	G	A	G	A	A	G	A	A	T	C	C	C	A	A	A	C	T	C	G
mx042	A	A	A	A	G	G	A	T	C	T	G	G	T	C	T	A	T	G	T	G	G	A	G	C	C	G	T	C	G	C	C	G	C	C	A	T	T	C	T	G	C	G	A	G	A	A	G	A	A	T	C	C	C	A	A	A	C	T	C	G
mx002	A	A	A	A	G	G	A	T	C	T	G	G	T	C	T	A	T	G	T	G	G	A	G	C	C	G	T	C	G	C	C	G	C	C	A	T	T	C	T	G	C	G	A	G	A	A	G	A	A	T	C	C	C	A	A	A	C	T	C	G
mx053	A	A	A	A	G	G	A	T	C	T	G	G	T	C	T	A	T	G	T	G	G	A	G	C	C	G	T	C	G	C	C	G	C	C	A	T	T	C	T	G	C	G	A	G	A	A	G	A	A	T	C	C	C	A	A	A	C	T	C	G
mx109	A	A	A	A	G	G	A	T	C	T	G	G	T	C	T	A	T	G	T	G	G	A	G	C	C	G	T	C	G	C	C	G	C	C	A	T	T	C	T	G	C	G	A	G	A	A	G	A	A	T	C	C	C	A	A	A	C	T	C	G
mx110	A	A	A	A	G	G	A	T	C	T	G	G	T	C	T	A	T	G	T	G	G	A	G	C	C	G	T	C	G	C	C	G	C	C	A	T	T	C	T	G	C	G	A	G	A	A	G	A	A	T	C	C	C	A	A	A	C	T	C	G
mx013	A	A	A	A	G	G	A	T	C	T	G	G	T	C	T	A	T	G	T	G	G	A	G	C	C	G	T	C	G	C	C	G	C	C	A	T	T	C	T	G	C	G	A	G	A	A	G	A	A	T	C	C	C	A	A	A	C	T	C	G
mx034	A	A	A	A	G	G	A	T	C	T	G	G	T	C	T	A	T	G	T	G	G	A	G	C	C	G	T	C	G	C	C	G	C	C	A	T	T	C	T	G	C	G	A	G	A	A	G	A	A	T	C	C	C	A	A	A	C	T	C	G
mx020	A	A	A	A	G	G	A	T	C	T	G	G	T	C	T	A	T	G	T	G	G	A	G	C	C	G	T	C	G	C	C	G	C	C	A	T	T	C	T	G	C	G	A	G	A	A	G	A	A	T	C	C	C	A	A	A	C	T	C	G
mx048	A	A	A	A	G	G	A	T	C	T	G	G	T	C	T	A	T	G	T	G	G	A	G	C	C	G	T	C	G	C	C	G	C	C	A	T	T	C	T	G	C	G	A	G	A	A	G	A	A	T	C	C	C	A	A	A	C	T	C	G
mx045	A	A	A	A	G	G	A	T	C	T	G	G	T	C	T	A	T	G	T	G	G	A	G	C	C	G	T	C	G	C	C	G	C	C	A	T	T	C	T	G	C	G	A	G	A	A	G	A	A	T	C	C	C	A	A	A	C	T	C	G
mx025	A	A	A	A	G	G	A	T	C	T	G	G	T	C	T	A	T	G	T	G	G	A	G	C	C	G	T	C	G	C	C	G	C	C	A	T	T	C	T	G	C	G	A	G	A	A	G	A	A	T	C	C	C	A	A	A	C	T	C	G
mx024	A	A	A	A	G	G	A	T	C	T	G	G	T	C	T	A	T	G	T	G	G	A	G	C	C	G	T	C	G	C	C	G	C	C	A	T	T	C	T	G	C	G	A	G	A	A	G	A	A	T	C	C	C	A	A	A	C	T	C	G
mx044	A	A	A	A	G	G	A	T	C	T	G	G	T	C	T	A	T	G	T	G	G	A	G	C	C	G	T	C	G	C	C	G	C	C	A	T	T	C	T	G	C	G	A	G	A	A	G	A	A	T	C	C	C	A	A	A	C	T	C	G
mx049	A	A	A	A	G	G	A	T	C	T	G	G	T	C	T	A	T	G	T	G	G	A	G	C	C	G	T	C	G	C	C	G	C	C	A	T	T	C	T	G	C	G	A	G	A	A	G	A	A	T	C	C	C	A	A	A	C	T	C	G
mx050	A	A	A	A	G	G	A	T	C	T	G	G	T	C	T	A	T	G	T	G	G	A	G	C	C	G	T	C	G	C	C	G	C	C	A	T	T	C	T	G	C	G	A	G	A	A	G	A	A	T	C	C	C	A	A	A	C	T	C	G
mx051	A	A	A	A	G	G	A	T	C	T	G	G	T	C	T	A	T	G	T	G	G	A	G	C	C	G	T	C	G	C	C	G	C	C	A	T	T	C	T	G	C	G	A	G	A	A	G	A	A	T	C	C	C	A	A	A	C	T	C	G
mx055	A	A	A	A	G	G	A	T	C	T	G	G	T	C	T	A	T	G	T	G	G	A	G	C	C	G	T	C	G	C	C	G	C	C	A	T	T	C	T	G	C	G	A	G	A	A	G	A	A	T	C	C	C	A	A	A	C	T	C	G
mx111	A	A	A	A	G	G	A	T	C	T	G	G	T	C	T	A	T	G	T	G	G	A	G	C	C	G	T	C	G	C	C	G	C	C	A	T	T	C	T	G	C	G	A	G	A	A	G	A	A	T	C	C	C	A	A	A	C	T	C	G
mx069	A	A	A	A	G	G	A	T	C	T	G	G	T	C	T	A	T	G	T	G	G	A	G	C	C	G	T	C	G	C	C	G	C	C	A	T	T	C	T	G	C	G	A	G	A	A	G	A	A	T	C	C	C	A	A	A	C	T	C	G
mx062	A	A	A	A	G	G	A	T	C	T	G	G	T	C	T	A	T	G	T	G	G	A	G	C	C	G	T	C	G	C	C	G	C	C	A	T	T	C	T	G	C	G	A	G	A	A	G	A	A	T	C	C	C	A	A	A	C	T	C	G
mx112	A	A	A	A	G	G	A	T	C	T	G	G	T	C	T	A	T	G	T	G	G	A	G	C	C	G	T	C	G	C	C	G	C	C	A	T	T	C	T	G	C	G	A	G	A	A	G	A	A	T	C	C	C	A	A	A	C	T	C	G
mx065	A	A	A	A	G	G	A	T	C	T	G	G	T	C	T	A	T	G	T	G	G	A	G	C	C	G	T	C	G	C	C	G	C	C	A	T	T	C	T	G	C	G	A	G	A	A	G	A	A	T	C	C	C	A	A	A	C	T	C	G
mx074	A	A	A	A	G	G	A	T	C	T	G	G	T	C	T	A	T	G	T	G	G	A	G	C	C	G	T	C	G	C	C	G	C	C	A	T	T	C	T	G	C	G	A	G	A	A	G	A	A	T	C	C	C	A	A	A	C	T	C	G
mx084	A	A	A	A	G	G	A	T	C	T	G	G	T	C	T	A	T	G	T	G	G	A	G	C	C	G	T	C	G	C	C	G	C	C	A	T	T	C	T	G	C	G	A	G	A	A	G	A	A	T	C	C	C	A	A	A	C	T	C	G
mx066	A	A	A	A	G	G	A	T	C	T	G	G	T	C	T	A	T	G	T	G	G	A	G	C	C	G	T	C	G	C	C	G	C	C	A	T	T	C	T	G	C	G	A	G	A	A	G	A	A	T	C	C	C	A	A	A	C	T	C	G
mx072	A	A	A	A	G	G	A	T	C	T	G	G	T	C	T	A	T	G	T	G	G	A	G	C	C	G	T	C	G	C	C	G	C	C	A	T	T	C	T	G	C	G	A	G	A	A	G	A	A	T	C	C	C	A	A	A	C	T	C	G
mx086	A	A	A	A	G	G	A	T	C	T	G	G	T	C	T	A	T	G	T	G	G	A	G	C	C	G	T	C	G	C	C	G	C	C	A	T	T	C	T	G	C	G	A	G	A	A	G	A	A	T	C	C	C	A	A	A	C	T	C	G
mx091	A	A	A	A	G	G	A	T	C	T	G	G	T	C	T	A	T	G	T	G	G	A	G	C	C	G	T	C	G	C	C	G	C	C	A	T	T	C	T	G	C	G	A	G	A	A	G	A	A	T	C	C	C	A	A	A	C	T	C	G
mx113	A	A	A	A	G	G	A	T	C	T	G	G	T	C	T	A	T	G	T	G	G	A	G	C	C	G	T	C	G	C	C	G	C	C	A	T	T	C	T	G	C	G	A	G	A	A	G	A	A	T	C	C	C	A	A	A	C	T	C	G
mx067	A	A	A	A	G	G	A	T	C	T	G	G	T	C	T	A	T	G	T	G	G	A	G	C	C	G	T	C	G	C	C	G	C	C	A	T	T	C	T	G	C	G	A	G	A	A	G	A	A	T	C	C	C	A	A	A	C	T	C	G
mx014	A	A	A	A	G	G	A	T	C	T	G	G	T	C	T	A	T	G	T	G	G	A	G	C	C	G	T	C	G	C	C	G	C	C	A	T	T	C	T	G	C	G	A	G	A	A	G	A	A	T	C	C	C	A	A	A	C	T	C	G
mx007	A	A	A	A	G	G	A	T	C	T	G	G	T	C	T	A	T	G	T	G	G	A	G	C	C	G	T	C	G	C	C	G	C	C	A	T	T	C	T	G	C	G	A	G	A	A	G	A	A	T	C	C	C	A	A	A	C	T	C	G
mx017	A	A	A	A	G	G	A	T	C	T	G	G	T	C	T	A	T	G	T	G	G	A	G	C	C	G	T	C	G	C	C	G	C	C	A	T	T	C	T	G	C	G	A	G	A	A	G	A	A	T	C	C	C	A	A	A	C	T	C	G
mx026	A	A	A	A	G	G	A	T	C	T	G	G	T	C	T	A	T	G	T	G	G	A	G	C	C	G	T	C	G	C	C	G	C	C	A	T	T	C	T	G	C	G	A	G	A	A	G	A	A	T	C	C	C	A	A	A	C	T	C	G
mx060	A	A	A	A	G	G	A	T	C	T	G	G	T	C	T	A	T	G	T	G	G	A	G	C	C	G	T	C	G	C	C	G	C	C	A	T	T	C	T	G	C	G	A	G	A	A	G	A	A	T	C	C	C	A	A	A	C	T	C	G
mx054	A	A	A	A	G	G	A	T	C	T	G	G	T	C	T	A	T	G	T	G	G	A	G	C	C	G	T	C	G	C	C	G	C	C	A	T	T	C	T	G	C	G	A	G	A	A	G	A	A	T	C	C	C	A	A	A	C	T	C	G
mx076	A	A	A	A	G	G	A	T	C	T	G	G	T	C	T	A	T	G	T	G	G	A	G	C	C	G	T	C	G	C	C	G	C	C	A	T	T	C	T	G	C	G	A	G	A	A	G	A	A	T	C	C	C	A	A	A	C	T	C	G
mx085	A	A	A	A	G	G	A	T	C	T	G	G	T	C	T	A	T	G	T	G	G	A	G	C	C	G	T	C	G	C	C	G	C	C	A	T	T	C	T	G	C	G	A	G	A	A	G	A	A	T	C	C	C	A	A	A	C	T	C	G
mx114	A	A	A	A	G	G	A	T	C	T	G	G	T	C	T	A	T	G	T	G	G	A	G	C	C	G	T	C	G	C	C	G	C	C	A	T	T	C	T	G	C	G	A	G	A	A	G	A	A	T	C	C	C	A	A	A	C	T	C	G
mx098	A	A	A	A	G	G	A	T	C	T	G	G	T	C	T	A	T	G	T	G	G	A	G	C	C	G	T	C	G	C	C	G	C	C	A	T	T	C	T	G	C	G	A	G	A	A	G	A	A	T	C	C	C	A	A	A	C	T	C	G
mx092	A	A	A	A	G	G	A	T	C	T	G	G	T	C	T	A	T	G	T	G	G	A	G	C	C	G	T	C	G	C	C	G	C	C	A	T	T	C	T	G	C	G	A	G	A	A	G	A	A	T	C	C	C	A	A	A	C	T	C	G
mx099	A	A	A	A	G	G	A	T	C	T	G	G	T	C	T	A	T	G	T	G	G	A	G	C	C	G	T	C	G	C	C	G	C	C	A	T	T	C	T	G	C	G	A	G	A	A	G	A	A	T	C	C	C	A	A	A	C	T	C	G
mx096	A	A	A	A	G	G	A	T	C	T	G	G	T	C	T	A	T	G	T	G	G	A	G	C	C	G	T	C	G	C	C	G	C	C	A	T	T	C	T	G	C	G	A	G	A	A	G	A	A	T	C	C	C	A	A	A	C	T	C	G
mx097	A	A	A	A	G	G	A	T	C	T	G	G	T	C	T	A	T	G	T	G	G	A	G	C	C	G	T	C	G	C	C	G	C	C	A	T	T	C	T	G	C	G	A	G	A	A	G	A	A	T	C	C	C	A	A	A	C	T	C	G
mx095	A	A	A	A	G	G	A	T	C	T	G	G	T	C	T	A	T	G	T	G	G	A	G	C	C	G	T	C	G	C	C	G	C	C	A	T	T	C	T	G	C	G	A	G	A	A	G	A	A	T	C	C	C	A	A	A	C	T	C	G
mx094	A	A	A	A	G	G	A	T	C	T	G	G	T	C	T	A	T	G	T	G	G	A	G	C	C	G	T	C	G	C	C	G	C	C	A	T	T	C	T	G	C	G	A	G	A	A	G	A	A	T	C	C	C	A	A	A	C	T	C	G

Continued

	241									250										260										270										280										290										300
mx001	G	C	A	T	C	C	T	G	G	G	C	A	C	T	C	A	C	G	G	G	C	G	A	C	A	G	T	G	C	A	A	C	G	A	C	A	C	G	A	G	C	A	T	C	G	G	C	G	T	T	G	A	T	G	G	C	T	G	C	G
mx087	G	C	A	T	C	C	T	G	G	G	C	A	C	T	C	A	C	G	G	G	C	G	A	C	A	G	T	G	C	A	A	C	G	A	C	A	C	G	A	G	C	A	T	C	G	G	C	G	T	T	G	A	T	G	G	C	T	G	C	G
mx035	G	C	A	T	C	C	T	G	G	G	C	A	C	T	C	A	C	G	G	G	C	G	A	C	A	G	T	G	C	A	A	C	G	A	C	A	C	G	A	G	C	A	T	C	G	G	C	G	T	T	G	A	T	G	G	C	T	G	C	G
mx004	G	C	A	T	C	C	T	G	G	G	C	A	C	T	C	A	C	G	G	G	C	G	A	C	A	G	T	G	C	A	A	C	G	A	C	A	C	G	A	G	C	A	T	C	G	G	C	G	T	T	G	A	T	G	G	C	T	G	C	G
mx008	G	C	A	T	C	C	T	G	G	G	C	A	C	T	C	A	C	G	G	G	C	G	A	C	A	G	T	G	C	A	A	C	G	A	C	A	C	G	A	G	C	A	T	C	G	G	C	G	T	T	G	A	T	G	G	C	T	G	C	G
mx042	G	C	A	T	C	C	T	G	G	G	C	A	C	T	C	A	C	G	G	G	C	G	A	C	A	G	T	G	C	A	A	C	G	A	C	A	C	G	A	G	C	A	T	C	G	G	C	G	T	T	G	A	T	G	G	C	T	G	C	G
mx002	G	C	A	T	C	C	T	G	G	G	C	A	C	T	C	A	C	G	G	G	C	G	A	C	A	G	T	G	C	A	A	C	G	A	C	A	C	G	A	G	C	A	T	C	G	G	C	G	T	T	G	A	T	G	G	C	T	G	C	G
mx053	G	C	A	T	C	C	T	G	G	G	C	A	C	T	C	A	C	G	G	G	C	G	A	C	A	G	T	G	C	A	A	C	G	A	C	A	C	G	A	G	C	A	T	C	G	G	C	G	T	T	G	A	T	G	G	C	T	G	C	G
mx109	G	C	A	T	C	C	T	G	G	G	C	A	C	T	C	A	C	G	G	G	C	G	A	C	A	G	T	G	C	A	A	C	G	A	C	A	C	G	A	G	C	A	T	C	G	G	C	G	T	T	G	A	T	G	G	C	T	G	C	G
mx110	G	C	A	T	C	C	T	G	G	G	C	A	C	T	C	A	C	G	G	G	C	G	A	C	A	G	T	G	C	A	A	C	G	A	C	A	C	G	A	G	C	A	T	C	G	G	C	G	T	T	G	A	T	G	G	C	T	G	C	G
mx013	G	C	A	T	C	C	T	G	G	G	C	A	C	T	C	A	C	G	G	G	C	G	A	C	A	G	T	G	C	A	A	C	G	A	C	A	C	G	A	G	C	A	T	C	G	G	C	G	T	T	G	A	T	G	G	C	T	G	C	G
mx034	G	C	A	T	C	C	T	G	G	G	C	A	C	T	C	A	C	G	G	G	C	G	A	C	A	G	T	G	C	A	A	C	G	A	C	A	C	G	A	G	C	A	T	C	G	G	C	G	T	T	G	A	T	G	G	C	T	G	C	G
mx020	G	C	A	T	C	C	T	G	G	G	C	A	C	T	C	A	C	G	G	G	C	G	A	C	A	G	T	G	C	A	A	C	G	A	C	A	C	G	A	G	C	A	T	C	G	G	C	G	T	T	G	A	T	G	G	C	T	G	C	G
mx048	G	C	A	T	C	C	T	G	G	G	C	A	C	T	C	A	C	G	G	G	C	G	A	C	A	G	T	G	C	A	A	C	G	A	C	A	C	G	A	G	C	A	T	C	G	G	C	G	T	T	G	A	T	G	G	C	T	G	C	G
mx045	G	C	A	T	C	C	T	G	G	G	C	A	C	T	C	A	C	G	G	G	C	G	A	C	A	G	T	G	C	A	A	C	G	A	C	A	C	G	A	G	C	A	T	C	G	G	C	G	T	T	G	A	T	G	G	C	T	G	C	G
mx025	G	C	A	T	C	C	T	G	G	G	C	A	C	T	C	A	C	G	G	G	C	G	A	C	A	G	T	G	C	A	A	C	G	A	C	A	C	G	A	G	C	A	T	C	G	G	C	G	T	T	G	A	T	G	G	C	T	G	C	G
mx024	G	C	A	T	C	C	T	G	G	G	C	A	C	T	C	A	C	G	G	G	C	G	A	C	A	G	T	G	C	A	A	C	G	A	C	A	C	G	A	G	C	A	T	C	G	G	C	G	T	T	G	A	T	G	G	C	T	G	C	G
mx044	G	C	A	T	C	C	T	G	G	G	C	A	C	T	C	A	C	G	G	G	C	G	A	C	A	G	T	G	C	A	A	C	G	A	C	A	C	G	A	G	C	A	T	C	G	G	C	G	T	T	G	A	T	G	G	C	T	G	C	G
mx049	G	C	A	T	C	C	T	G	G	G	C	A	C	T	C	A	C	G	G	G	C	G	A	C	A	G	T	G	C	A	A	C	G	A	C	A	C	G	A	G	C	A	T	C	G	G	C	G	T	T	G	A	T	G	G	C	T	G	C	G
mx050	G	C	A	T	C	C	T	G	G	G	C	A	C	T	C	A	C	G	G	G	C	G	A	C	A	G	T	G	C	A	A	C	G	A	C	A	C	G	A	G	C	A	T	C	G	G	C	G	T	T	G	A	T	G	G	C	T	G	C	G
mx051	G	C	A	T	C	C	T	G	G	G	C	A	C	T	C	A	C	G	G	G	C	G	A	C	A	G	T	G	C	A	A	C	G	A	C	A	C	G	A	G	C	A	T	C	G	G	C	G	T	T	G	A	T	G	G	C	T	G	C	G
mx055	G	C	A	T	C	C	T	G	G	G	C	A	C	T	C	A	C	G	G	G	C	G	A	C	A	G	T	G	C	A	A	C	G	A	C	A	C	G	A	G	C	A	T	C	G	G	C	G	T	T	G	A	T	G	G	C	T	G	C	G
mx111	G	C	A	T	C	C	T	G	G	G	C	A	C	T	C	A	C	G	G	G	C	G	A	C	A	G	T	G	C	A	A	C	G	A	C	A	C	G	A	G	C	A	T	C	G	G	C	G	T	T	G	A	T	G	G	C	T	G	C	G
mx069	G	C	A	T	C	C	T	G	G	G	C	A	C	T	C	A	C	G	G	G	C	G	A	C	A	G	T	G	C	A	A	C	G	A	C	A	C	G	A	G	C	A	T	C	G	G	C	G	T	T	G	A	T	G	G	C	T	G	C	G
mx062	G	C	A	T	C	C	T	G	G	G	C	A	C	T	C	A	C	G	G	G	C	G	A	C	A	G	T	G	C	A	A	C	G	A	C	A	C	G	A	G	C	A	T	C	G	G	C	G	T	T	G	A	T	G	G	C	T	G	C	G
mx112	G	C	A	T	C	C	T	G	G	G	C	A	C	T	C	A	C	G	G	G	C	G	A	C	A	G	T	G	C	A	A	C	G	A	C	A	C	G	A	G	C	A	T	C	G	G	C	G	T	T	G	A	T	G	G	C	T	G	C	G
mx065	G	C	A	T	C	C	T	G	G	G	C	A	C	T	C	A	C	G	G	G	C	G	A	C	A	G	T	G	C	A	A	C	G	A	C	A	C	G	A	G	C	A	T	C	G	G	C	G	T	T	G	A	T	G	G	C	T	G	C	G
mx074	G	C	A	T	C	C	T	G	G	G	C	A	C	T	C	A	C	G	G	G	C	G	A	C	A	G	T	G	C	A	A	C	G	A	C	A	C	G	A	G	C	A	T	C	G	G	C	G	T	T	G	A	T	G	G	C	T	G	C	G
mx084	G	C	A	T	C	C	T	G	G	G	C	A	C	T	C	A	C	G	G	G	C	G	A	C	A	G	T	G	C	A	A	C	G	A	C	A	C	G	A	G	C	A	T	C	G	G	C	G	T	T	G	A	T	G	G	C	T	G	C	G
mx066	G	C	A	T	C	C	T	G	G	G	C	A	C	T	C	A	C	G	G	G	C	G	A	C	A	G	T	G	C	A	A	C	G	A	C	A	C	G	A	G	C	A	T	C	G	G	C	G	T	T	G	A	T	G	G	C	T	G	C	G
mx072	G	C	A	T	C	C	T	G	G	G	C	A	C	T	C	A	C	G	G	G	C	G	A	C	A	G	T	G	C	A	A	C	G	A	C	A	C	G	A	G	C	A	T	C	G	G	C	G	T	T	G	A	T	G	G	C	T	G	C	G
mx086	G	C	A	T	C	C	T	G	G	G	C	A	C	T	C	A	C	G	G	G	C	G	A	C	A	G	T	G	C	A	A	C	G	A	C	A	C	G	A	G	C	A	T	C	G	G	C	G	T	T	G	A	T	G	G	C	T	G	C	G
mx091	G	C	A	T	C	C	T	G	G	G	C	A	C	T	C	A	C	G	G	G	C	G	A	C	A	G	T	G	C	A	A	C	G	A	C	A	C	G	A	G	C	A	T	C	G	G	C	G	T	T	G	A	T	G	G	C	T	G	C	G
mx113	G	C	A	T	C	C	T	G	G	G	C	A	C	T	C	A	C	G	G	G	C	G	A	C	A	G	T	G	C	A	A	C	G	A	C	A	C	G	A	G	C	A	T	C	G	G	C	G	T	T	G	A	T	G	G	C	T	G	C	G
mx067	G	C	A	T	C	C	T	G	G	G	C	A	C	T	C	A	C	G	G	G	C	G	A	C	A	G	T	G	C	A	A	C	G	A	C	A	C	G	A	G	C	A	T	C	G	G	C	G	T	T	G	A	T	G	G	C	T	G	C	G
mx014	G	C	A	T	C	C	T	G	G	G	C	A	C	T	C	A	C	G	G	G	C	G	A	C	A	G	T	G	C	A	A	C	G	A	C	A	C	G	A	G	C	A	T	C	G	G	C	G	T	T	G	A	T	G	G	C	T	G	C	G
mx007	G	C	A	T	C	C	T	G	G	G	C	A	C	T	C	A	C	G	G	G	C	G	A	C	A	G	T	G	C	A	A	C	G	A	C	A	C	G	A	G	C	A	T	C	G	G	C	G	T	T	G	A	T	G	G	C	T	G	C	G
mx017	G	C	A	T	C	C	T	G	G	G	C	A	C	T	C	A	C	G	G	G	C	G	A	C	A	G	T	G	C	A	A	C	G	A	C	A	C	G	A	G	C	A	T	C	G	G	C	G	T	T	G	A	T	G	G	C	T	G	C	G
mx026	G	C	A	T	C	C	T	G	G	G	C	A	C	T	C	A	C	G	G	G	C	G	A	C	A	G	T	G	C	A	A	C	G	A	C	A	C	G	A	G	C	A	T	C	G	G	C	G	T	T	G	A	T	G	G	C	T	G	C	G
mx060	G	C	A	T	C	C	T	G	G	G	C	A	C	T	C	A	C	G	G	G	C	G	A	C	A	G	T	G	C	A	A	C	G	A	C	A	C	G	A	G	C	A	T	C	G	G	C	G	T	T	G	A	T	G	G	C	T	G	C	G
mx054	G	C	A	T	C	C	T	G	G	G	C	A	C	T	C	A	C	G	G	G	C	G	A	C	A	G	T	G	C	A	A	C	G	A	C	A	C	G	A	G	C	A	T	C	G	G	C	G	T	T	G	A	T	G	G	C	T	G	C	G
mx076	G	C	A	T	C	C	T	G	G	G	C	A	C	T	C	A	C	G	G	G	C	G	A	C	A	G	T	G	C	A	A	C	G	A	C	A	C	G	A	G	C	A	T	C	G	G	C	G	T	T	G	A	T	G	G	C	T	G	C	G
mx085	G	C	A	T	C	C	T	G	G	G	C	A	C	T	C	A	C	G	G	G	C	G	A	C	A	G	T	G	C	A	A	C	G	A	C	A	C	G	A	G	C	A	T	C	G	G	C	G	T	T	G	A	T	G	G	C	T	G	C	G
mx114	G	C	A	T	C	C	T	G	G	G	C	A	C	T	C	A	C	G	G	G	C	G	A	C	A	G	T	G	C	A	A	C	G	A	C	A	C	G	A	G	C	A	T	C	G	G	C	G	T	T	G	A	T	G	G	C	T	G	C	G
mx098	G	C	A	T	C	C	T	G	G	G	C	A	C	T	C	A	C	G	G	G	C	G	A	C	A	G	T	G	C	A	A	C	G	A	C	A	C	G	A	G	C	A	T	C	G	G	C	G	T	T	G	A	T	G	G	C	T	G	C	G
mx092	G	C	A	T	C	C	T	G	G	G	C	A	C	T	C	A	C	G	G	G	C	G	A	C	A	G	T	G	C	A	A	C	G	A	C	A	C	G	A	G	C	A	T	C	G	G	C	G	T	T	G	A	T	G	G	C	T	G	C	G
mx099	G	C	A	T	C	C	T	G	G	G	C	A	C	T	C	A	C	G	G	G	C	G	A	C	A	G	T	G	C	A	A	C	G	A	C	A	C	G	A	G	C	A	T	C	G	G	C	G	T	T	G	A	T	G	G	C	T	G	C	G
mx096	G	C	A	T	C	C	T	G	G	G	C	A	C	T	C	A	C	G	G	G	C	G	A	C	A	G	T	G	C	A	A	C	G	A	C	A	C	G	A	G	C	A	T	C	G	G	C	G	T	T	G	A	T	G	G	C	T	G	C	G
mx097	G	C	A	T	C	C	T	G	G	G	C	A	C	T	C	A	C	G	G	G	C	G	A	C	A	G	T	G	C	A	A	C	G	A	C	A	C	G	A	G	C	A	T	C	G	G	C	G	T	T	G	A	T	G	G	C	T	G	C	G
mx095	G	C	A	T	C	C	T	G	G	G	C	A	C	T	C	A	C	G	G	G	C	G	A	C	A	G	T	G	C	A	A	C	G	A	C	A	C	G	A	G	C	A	T	C	G	G	C	G	T	T	G	A	T	G	G	C	T	G	C	G
mx094	G	C	A	T	C	C	T	G	G	G	C	A	C	T	C	A	C	G	G	G	C	G	A	C	A	G	T	G	C	A	A	C	G	A	C	A	C	G	A	G	C	A	T	C	G	G	C	G	T	T	G	A	T	G	G	C	T	G	C	G

Continued

	301									310										320										330										340										350										360
mx001	A	C	C	T	G	A	T	G	T	G	C	T	G	C	G	G	T	A	G	G	G	G	T	C	A	C	A	A	G	A	C	T	C	A	G	G	A	G	G	T	G	A	C	G	G	T	A	A	T	C	G	A	G	A	G	G	T	G	C	T
mx087	A	C	C	T	G	A	T	G	T	G	C	T	G	C	G	G	T	A	G	G	G	G	T	C	A	C	A	A	G	A	C	T	C	A	G	G	A	G	G	T	G	A	C	G	G	T	A	A	T	C	G	A	G	A	G	G	T	G	C	T
mx035	A	C	C	T	G	A	T	G	T	G	C	T	G	C	G	G	T	A	G	G	G	G	T	C	A	C	A	A	G	A	C	T	C	A	G	G	A	G	G	T	G	A	C	G	G	T	A	A	T	C	G	A	G	A	G	G	T	G	C	T
mx004	A	C	C	T	G	A	T	G	T	G	C	T	G	C	G	G	T	A	G	G	G	G	T	C	A	C	A	A	G	A	C	T	C	A	G	G	A	G	G	T	G	A	C	G	G	T	A	A	T	C	G	A	G	A	G	G	T	G	C	T
mx008	A	C	C	T	G	A	T	G	T	G	C	T	G	C	G	G	T	A	G	G	G	G	T	C	A	C	A	A	G	A	C	T	C	A	G	G	A	G	G	T	G	A	C	G	G	T	A	A	T	C	G	A	G	A	G	G	T	G	C	T
mx042	A	C	C	T	G	A	T	G	T	G	C	T	G	C	G	G	T	A	G	G	G	G	T	C	A	C	A	A	G	A	C	T	C	A	G	G	A	G	G	T	G	A	C	G	G	T	A	A	T	C	G	A	G	A	G	G	T	G	C	T
mx002	A	C	C	T	G	A	T	G	T	G	C	T	G	C	G	G	T	A	G	G	G	G	T	C	A	C	A	A	G	A	C	T	C	A	G	G	A	G	G	T	G	A	C	G	G	T	A	A	T	C	G	A	G	A	G	G	T	G	C	T
mx053	A	C	C	T	G	A	T	G	T	G	C	T	G	C	G	G	T	A	G	G	G	G	T	C	A	C	A	A	G	A	C	T	C	A	G	G	A	G	G	T	G	A	C	G	G	T	A	A	T	C	G	A	G	A	G	G	T	G	C	T
mx109	A	C	C	T	G	A	T	G	T	G	C	T	G	C	G	G	T	A	G	G	G	G	T	C	A	C	A	A	G	A	C	T	C	A	G	G	A	G	G	T	G	A	C	G	G	T	A	A	T	C	G	A	G	A	G	G	T	G	C	T
mx110	A	C	C	T	G	A	T	G	T	G	C	T	G	C	G	G	T	A	G	G	G	G	T	C	A	C	A	A	G	A	C	T	C	A	G	G	A	G	G	T	G	A	C	G	G	T	A	A	T	C	G	A	G	A	G	G	T	G	C	T
mx013	A	C	C	T	G	A	T	G	T	G	C	T	G	C	G	G	T	A	G	G	G	G	T	C	A	C	A	A	G	A	C	T	C	A	G	G	A	G	G	T	G	A	C	G	G	T	A	A	T	C	G	A	G	A	G	G	T	G	C	T
mx034	A	C	C	T	G	A	T	G	T	G	C	T	G	C	G	G	T	A	G	G	G	G	T	C	A	C	A	A	G	A	C	T	C	A	G	G	A	G	G	T	G	A	C	G	G	T	A	A	T	C	G	A	G	A	G	G	T	G	C	T
mx020	A	C	C	T	G	A	T	G	T	G	C	T	G	C	G	G	T	A	G	G	G	G	T	C	A	C	A	A	G	A	C	T	C	A	G	G	A	G	G	T	G	A	C	G	G	T	A	A	T	C	G	A	G	A	G	G	T	G	C	T
mx048	A	C	C	T	G	A	T	G	T	G	C	T	G	C	G	G	T	A	G	G	G	G	T	C	A	C	A	A	G	A	C	T	C	A	G	G	A	G	G	T	G	A	C	G	G	T	A	A	T	C	G	A	G	A	G	G	T	G	C	T
mx045	A	C	C	T	G	A	T	G	T	G	C	T	G	C	G	G	T	A	G	G	G	G	T	C	A	C	A	A	G	A	C	T	C	A	G	G	A	G	G	T	G	A	C	G	G	T	A	A	T	C	G	A	G	A	G	G	T	G	C	T
mx025	A	C	C	T	G	A	T	G	T	G	C	T	G	C	G	G	T	A	G	G	G	G	T	C	A	C	A	A	G	A	C	T	C	A	G	G	A	G	G	T	G	A	C	G	G	T	A	A	T	C	G	A	G	A	G	G	T	G	C	T
mx024	A	C	C	T	G	A	T	G	T	G	C	T	G	C	G	G	T	A	G	G	G	G	T	C	A	C	A	A	G	A	C	T	C	A	G	G	A	G	G	T	G	A	C	G	G	T	A	A	T	C	G	A	G	A	G	G	T	G	C	T
mx044	A	C	C	T	G	A	T	G	T	G	C	T	G	C	G	G	T	A	G	G	G	G	T	C	A	C	A	A	G	A	C	T	C	A	G	G	A	G	G	T	G	A	C	G	G	T	A	A	T	C	G	A	G	A	G	G	T	G	C	T
mx049	A	C	C	T	G	A	T	G	T	G	C	T	G	C	G	G	T	A	G	G	G	G	T	C	A	C	A	A	G	A	C	T	C	A	G	G	A	G	G	T	G	A	C	G	G	T	A	A	T	C	G	A	G	A	G	G	T	G	C	T
mx050	A	C	C	T	G	A	T	G	T	G	C	T	G	C	G	G	T	A	G	G	G	G	T	C	A	C	A	A	G	A	C	T	C	A	G	G	A	G	G	T	G	A	C	G	G	T	A	A	T	C	G	A	G	A	G	G	T	G	C	T
mx051	A	C	C	T	G	A	T	G	T	G	C	T	G	C	G	G	T	A	G	G	G	G	T	C	A	C	A	A	G	A	C	T	C	A	G	G	A	G	G	T	G	A	C	G	G	T	A	A	T	C	G	A	G	A	G	G	T	G	C	T
mx055	A	C	C	T	G	A	T	G	T	G	C	T	G	C	G	G	T	A	G	G	G	G	T	C	A	C	A	A	G	A	C	T	C	A	G	G	A	G	G	T	G	A	C	G	G	T	A	A	T	C	G	A	G	A	G	G	T	G	C	T
mx111	A	C	C	T	G	A	T	G	T	G	C	T	G	C	G	G	T	A	G	G	G	G	T	C	A	C	A	A	G	A	C	T	C	A	G	G	A	G	G	T	G	A	C	G	G	T	A	A	T	C	G	A	G	A	G	G	T	G	C	T
mx069	A	C	C	T	G	A	T	G	T	G	C	T	G	C	G	G	T	A	G	G	G	G	T	C	A	C	A	A	G	A	C	T	C	A	G	G	A	G	G	T	G	A	C	G	G	T	A	A	T	C	G	A	G	A	G	G	T	G	C	T
mx062	A	C	C	T	G	A	T	G	T	G	C	T	G	C	G	G	T	A	G	G	G	G	T	C	A	C	A	A	G	A	C	T	C	A	G	G	A	G	G	T	G	A	C	G	G	T	A	A	T	C	G	A	G	A	G	G	T	G	C	T
mx112	A	C	C	T	G	A	T	G	T	G	C	T	G	C	G	G	T	A	G	G	G	G	T	C	A	C	A	A	G	A	C	T	C	A	G	G	A	G	G	T	G	A	C	G	G	T	A	A	T	C	G	A	G	A	G	G	T	G	C	T
mx065	A	C	C	T	G	A	T	G	T	G	C	T	G	C	G	G	T	A	G	G	G	G	T	C	A	C	A	A	G	A	C	T	C	A	G	G	A	G	G	T	G	A	C	G	G	T	A	A	T	C	G	A	G	A	G	G	T	G	C	T
mx074	A	C	C	T	G	A	T	G	T	G	C	T	G	C	G	G	T	A	G	G	G	G	T	C	A	C	A	A	G	A	C	T	C	A	G	G	A	G	G	T	G	A	C	G	G	T	A	A	T	C	G	A	G	A	G	G	T	G	C	T
mx084	A	C	C	T	G	A	T	G	T	G	C	T	G	C	G	G	T	A	G	G	G	G	T	C	A	C	A	A	G	A	C	T	C	A	G	G	A	G	G	T	G	A	C	G	G	T	A	A	T	C	G	A	G	A	G	G	T	G	C	T
mx066	A	C	C	T	G	A	T	G	T	G	C	T	G	C	G	G	T	A	G	G	G	G	T	C	A	C	A	A	G	A	C	T	C	A	G	G	A	G	G	T	G	A	C	G	G	T	A	A	T	C	G	A	G	A	G	G	T	G	C	T
mx072	A	C	C	T	G	A	T	G	T	G	C	T	G	C	G	G	T	A	G	G	G	G	T	C	A	C	A	A	G	A	C	T	C	A	G	G	A	G	G	T	G	A	C	G	G	T	A	A	T	C	G	A	G	A	G	G	T	G	C	T
mx086	A	C	C	T	G	A	T	G	T	G	C	T	G	C	G	G	T	A	G	G	G	G	T	C	A	C	A	A	G	A	C	T	C	A	G	G	A	G	G	T	G	A	C	G	G	T	A	A	T	C	G	A	G	A	G	G	T	G	C	T
mx091	A	C	C	T	G	A	T	G	T	G	C	T	G	C	G	G	T	A	G	G	G	G	T	C	A	C	A	A	G	A	C	T	C	A	G	G	A	G	G	T	G	A	C	G	G	T	A	A	T	C	G	A	G	A	G	G	T	G	C	T
mx113	A	C	C	T	G	A	T	G	T	G	C	T	G	C	G	G	T	A	G	G	G	G	T	C	A	C	A	A	G	A	C	T	C	A	G	G	A	G	G	T	G	A	C	G	G	T	A	A	T	C	G	A	G	A	G	G	T	G	C	T
mx067	A	C	C	T	G	A	T	G	T	G	C	T	G	C	G	G	T	A	G	G	G	G	T	C	A	C	A	A	G	A	C	T	C	A	G	G	A	G	G	T	G	A	C	G	G	T	A	A	T	C	G	A	G	A	G	G	T	G	C	T
mx014	A	C	C	T	G	A	T	G	T	G	C	T	G	C	G	G	T	A	G	G	G	G	T	C	A	C	A	A	G	A	C	T	C	A	G	G	A	G	G	T	G	A	C	G	G	T	A	A	T	C	G	A	G	A	G	G	T	G	C	T
mx007	A	C	C	T	G	A	T	G	T	G	C	T	G	C	G	G	T	A	G	G	G	G	T	C	A	C	A	A	G	A	C	T	C	A	G	G	A	G	G	T	G	A	C	G	G	T	A	A	T	C	G	A	G	A	G	G	T	G	C	T
mx017	A	C	C	T	G	A	T	G	T	G	C	T	G	C	G	G	T	A	G	G	G	G	T	C	A	C	A	A	G	A	C	T	C	A	G	G	A	G	G	T	G	A	C	G	G	T	A	A	T	C	G	A	G	A	G	G	T	G	C	T
mx026	A	C	C	T	G	A	T	G	T	G	C	T	G	C	G	G	T	A	G	G	G	G	T	C	A	C	A	A	G	A	C	T	C	A	G	G	A	G	G	T	G	A	C	G	G	T	A	A	T	C	G	A	G	A	G	G	T	G	C	T
mx060	A	C	C	T	G	A	T	G	T	G	C	T	G	C	G	G	T	A	G	G	G	G	T	C	A	C	A	A	G	A	C	T	C	A	G	G	A	G	G	T	G	A	C	G	G	T	A	A	T	C	G	A	G	A	G	G	T	G	C	T
mx054	A	C	C	T	G	A	T	G	T	G	C	T	G	C	G	G	T	A	G	G	G	G	T	C	A	C	A	A	G	A	C	T	C	A	G	G	A	G	G	T	G	A	C	G	G	T	A	A	T	C	G	A	G	A	G	G	T	G	C	T
mx076	A	C	C	T	G	A	T	G	T	G	C	T	G	C	G	G	T	A	G	G	G	G	T	C	A	C	A	A	G	A	C	T	C	A	G	G	A	G	G	T	G	A	C	G	G	T	A	A	T	C	G	A	G	A	G	G	T	G	C	T
mx085	A	C	C	T	G	A	T	G	T	G	C	T	G	C	G	G	T	A	G	G	G	G	T	C	A	C	A	A	G	A	C	T	C	A	G	G	A	G	G	T	G	A	C	G	G	T	A	A	T	C	G	A	G	A	G	G	T	G	C	T
mx114	A	C	C	T	G	A	T	G	T	G	C	T	G	C	G	G	T	A	G	G	G	G	T	C	A	C	A	A	G	A	C	T	C	A	G	G	A	G	G	T	G	A	C	G	G	T	A	A	T	C	G	A	G	A	G	G	T	G	C	T
mx098	A	C	C	T	G	A	T	G	T	G	C	T	G	C	G	G	T	A	G	G	G	G	T	C	A	C	A	A	G	A	C	T	C	A	G	G	A	G	G	T	G	A	C	G	G	T	A	A	T	C	G	A	G	A	G	G	T	G	C	T
mx092	A	C	C	T	G	A	T	G	T	G	C	T	G	C	G	G	T	A	G	G	G	G	T	C	A	C	A	A	G	A	C	T	C	A	G	G	A	G	G	T	G	A	C	G	G	T	A	A	T	C	G	A	G	A	G	G	T	G	C	T
mx099	A	C	C	T	G	A	T	G	T	G	C	T	G	C	G	G	T	A	G	G	G	G	T	C	A	C	A	A	G	A	C	T	C	A	G	G	A	G	G	T	G	A	C	G	G	T	A	A	T	C	G	A	G	A	G	G	T	G	C	T
mx096	A	C	C	T	G	A	T	G	T	G	C	T	G	C	G	G	T	A	G	G	G	G	T	C	A	C	A	A	G	A	C	T	C	A	G	G	A	G	G	T	G	A	C	G	G	T	A	A	T	C	G	A	G	A	G	G	T	G	C	T
mx097	A	C	C	T	G	A	T	G	T	G	C	T	G	C	G	G	T	A	G	G	G	G	T	C	A	C	A	A	G	A	C	T	C	A	G	G	A	G	G	T	G	A	C	G	G	T	A	A	T	C	G	A	G	A	G	G	T	G	C	T
mx095	A	C	C	T	G	A	T	G	T	G	C	T	G	C	G	G	T	A	G	G	G	G	T	C	A	C	A	A	G	A	C	T	C	A	G	G	A	G	G	T	G	A	C	G	G	T	A	A	T	C	G	A	G	A	G	G	T	G	C	T
mx094	A	C	C	T	G	A	T	G	T	G	C	T	G	C	G	G	T	A	G	G	G	G	T	C	A	C	A	A	G	A	C	T	C	A	G	G	A	G	G	T	G	A	C	G	G	T	A	A	T	C	G	A	G	A	G	G	T	G	C	T

Continued

	361									370		
mx001	C	C	T	G	C	A	C	T	T	T	C	C
mx087	C	C	T	G	C	A	C	T	T	T	C	C
mx035	C	C	T	G	C	A	C	T	T	T	C	C
mx004	C	C	T	G	C	A	C	T	T	T	C	C
mx008	C	C	T	G	C	A	C	T	T	T	C	C
mx042	C	C	T	G	C	A	C	T	T	T	C	C
mx002	C	C	T	G	C	A	C	T	T	T	C	C
mx053	C	C	T	G	C	A	C	T	T	T	C	C
mx109	C	C	T	G	C	A	C	T	T	T	C	C
mx110	C	C	T	G	C	A	C	T	T	T	C	C
mx013	C	C	T	G	C	A	C	T	T	T	C	C
mx034	C	C	T	G	C	A	C	T	T	T	C	C
mx020	C	C	T	G	C	A	C	T	T	T	C	C
mx048	C	C	T	G	C	A	C	T	T	T	C	C
mx045	C	C	T	G	C	A	C	T	T	T	C	C
mx025	C	C	T	G	C	A	C	T	T	T	C	C
mx024	C	C	T	G	C	A	C	T	T	T	C	C
mx044	C	C	T	G	C	A	C	T	T	T	C	C
mx049	C	C	T	G	C	A	C	T	T	T	C	C
mx050	C	C	T	G	C	A	C	T	T	T	C	C
mx051	C	C	T	G	C	A	C	T	T	T	C	C
mx055	C	C	T	G	C	A	C	T	T	T	C	C
mx111	C	C	T	G	C	A	C	T	T	T	C	C
mx069	C	C	T	G	C	A	C	T	T	T	C	C
mx062	C	C	T	G	C	A	C	T	T	T	C	C
mx112	C	C	T	G	C	A	C	T	T	T	C	C
mx065	C	C	T	G	C	A	C	T	T	T	C	C
mx074	C	C	T	G	C	A	C	T	T	T	C	C
mx084	C	C	T	G	C	A	C	T	T	T	C	C
mx066	C	C	T	G	C	A	C	T	T	T	C	C
mx072	C	C	T	G	C	A	C	T	T	T	C	C
mx086	C	C	T	G	C	A	C	T	T	T	C	C
mx091	C	C	T	G	C	A	C	T	T	T	C	C
mx113	C	C	T	G	C	A	C	T	T	T	C	C
mx067	C	C	T	G	C	A	C	T	T	T	C	C
mx014	C	C	T	G	C	A	C	T	T	T	C	C
mx007	C	C	T	G	C	A	C	T	T	T	C	C
mx017	C	C	T	G	C	A	C	T	T	T	C	C
mx026	C	C	T	G	C	A	C	T	T	T	C	C
mx060	C	C	T	G	C	A	C	T	T	T	C	C
mx054	C	C	T	G	C	A	C	T	T	T	C	C
mx076	C	C	T	G	C	A	C	T	T	T	C	C
mx085	C	C	T	G	C	A	C	T	T	T	C	C
mx114	C	C	T	G	C	A	C	T	T	T	C	C
mx098	C	C	T	G	C	A	C	T	T	T	C	C
mx092	C	C	T	G	C	A	C	T	T	T	C	C
mx099	C	C	T	G	C	A	C	T	T	T	C	C
mx096	C	C	T	G	C	A	C	T	T	T	C	C
mx097	C	C	T	G	C	A	C	T	T	T	C	C
mx095	C	C	T	G	C	A	C	T	T	T	C	C
mx094	C	C	T	G	C	A	C	T	T	T	C	C
