# Supplementary material for: Phylogeography of the sand dune ant Mycetophylax simplex along the Brazilian Atlantic Forest coast: remarkably low mtDNA diversity and shallow population structure
Source: BMC Evol Biol. 2015 Jun 10;15:106. doi: 10.1186/s12862-015-0383-4 (PMC4460702; doi:10.1186/s12862-015-0383-4)

Appendix S2 - Bayesian phylogenetic consensus tree of COI gene sequences of *M. simplex* haplotypes and out-groups. The numbers at branches are Bayesian posterior probabilities (P.P.). Three branches are proportional to mutational events sampled in sequences alignment (scale bar).

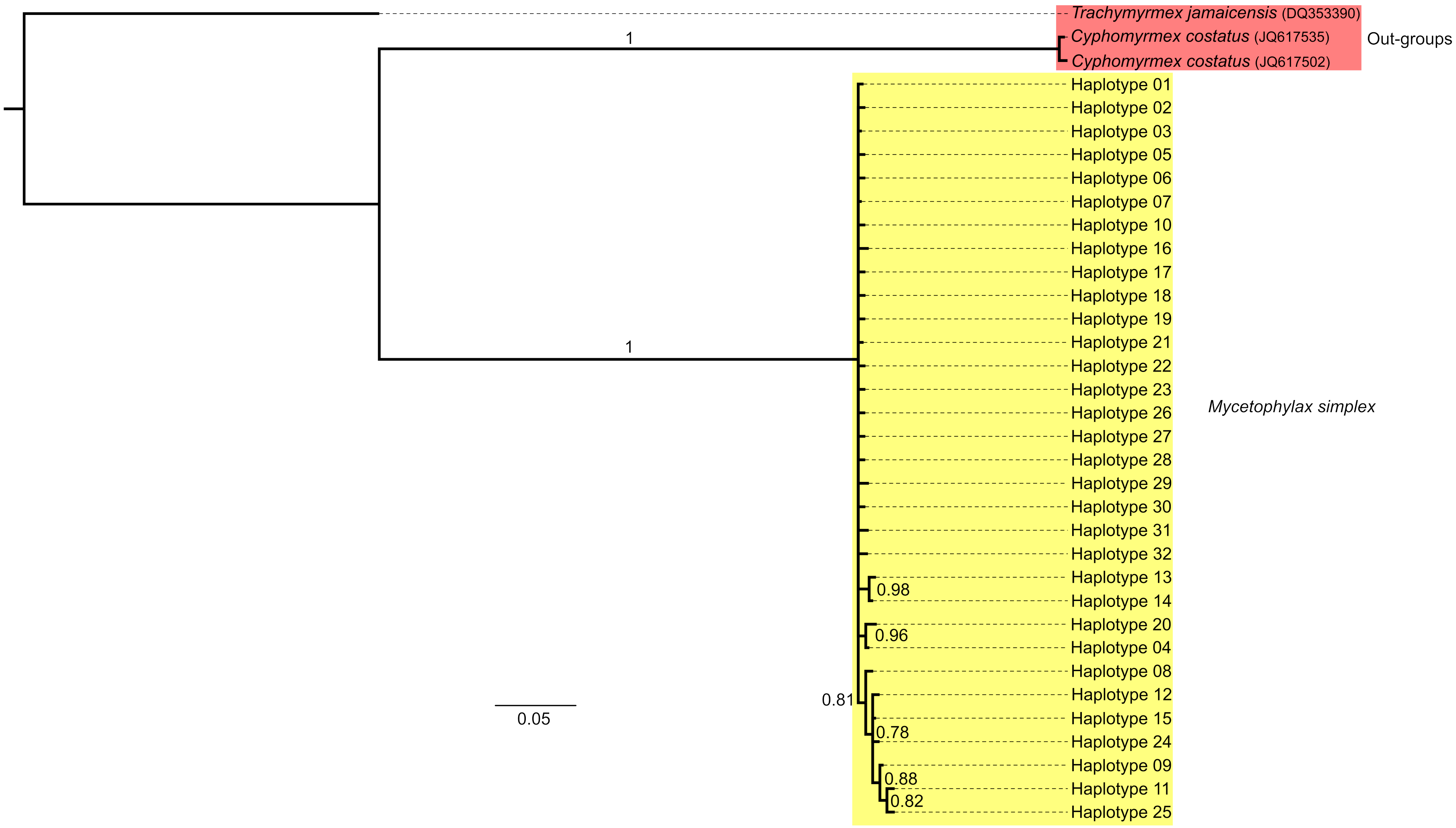

Supplement: Additional file 2: Appendix S2. — Bayesian phylogenetic consensus tree of COI gene sequences of M. simplex haplotypes and outgroups. The numbers at branches are Bayesian posterior probabilities (P.P.). Tree branches are proportional to mutational events sampled in sequences alignment (scale bar). [file 12862_2015_383_MOESM2_ESM.pdf]
